# Nanolipoprotein particle (NLP) vaccine confers protection against *Yersinia pestis* aerosol challenge in a BALB/c mouse model

**DOI:** 10.3389/fimmu.2025.1603710

**Published:** 2025-06-26

**Authors:** Sergei S. Biryukov, Amy Rasley, Michael L. Davies, Christopher P. Klimko, Jennifer L. Dankmeyer, Melissa Hunter, Nathaniel O. Rill, Jennifer L. Shoe, Jeremy Miller, Yuli Talyansky, Barbara Sullinger, Matheo Herrera, Daniel Huang, Leslie Bautista, Lucy Pepe, Sandra K. G. Peters, Christian J. Xander, Elsie E. Martinez, Ronald G. Toothman, Kevin D. Mlynek, Joel A. Bozue, Ju Qiu, Nicholas O. Fischer, Christopher K. Cote

**Affiliations:** ^1^ Bacteriology Division, United States Army Medical Research Institute of Infectious Diseases, Frederick, MD, United States; ^2^ Biosciences and Biotechnology Division, Lawrence Livermore National Laboratory, Livermore, CA, United States; ^3^ Department of Molecular and Cell Biology, University of California, Merced, Merced, United States; ^4^ Vaxcyte, Inc., San Carlos, CA, United States; ^5^ Regulated Research Administration: Biostatistics Division, United States Army Medical Research Institute of Infectious Diseases, Frederick, MD, United States

**Keywords:** plague, *Yersinia pestis*, vaccine, nanolipoprotein particle, mice, pneumonic, F1, LcrV

## Abstract

**Introduction:**

*Yersinia pestis* is the etiological agent of plague, a disease that remains a concern as demonstrated by recent outbreaks in Madagascar. Infection with *Y. pestis* results in a rapidly progressing illness that can only be successfully treated with antibiotics given shortly after symptom onset. Live attenuated or whole cell inactivated vaccines confer protection against bubonic plague, but pneumonic plague has been more difficult to prevent. Novel effective subunit vaccine formulations may circumvent some of these shortfalls. Here, we compare the immunogenicity generated by an advanced subunit vaccine (F1V fusion protein) and a nanolipoprotein particle (NLP)-based vaccine.

**Methods:**

The NLP, a high-density lipoprotein mimetic, provides a nanoscale delivery platform for recombinant *Y. pestis* antigens LcrV (V) and F1. BALB/c mice were immunized via subcutaneous injection twice, three or four weeks apart. Four weeks later, splenocytes and sera were collected for immune profiling, and mice were challenged with aerosolized *Y. pestis* CO92.

**Results:**

Both formulations induced a strong IgG response against the F1 and V proteins, along with a robust memory B cell response and a balanced cell-mediated immune response as evidenced by both Th1- and Th2-related cytokines. The NLP-based vaccine induced a stronger cytokine response against F1, V, and F1V proteins relative to the F1V vaccine. As with F1V, the inclusion of Alhydrogel (Alu) in NLP vaccine formulations was critical for enhanced immunogenicity and protective efficacy. Mice that received two doses of F1:V:NLP + Alu and CpG were completely protected from a challenge with approximately eight median lethal doses of aerosolized *Y. pestis* CO92 and this protection confirmed the well-documented synergy between the F1 and V antigens in context of pneumonic plague. The NLPs have defined regions of polarity that facilitates the incorporation of a wide range of adjuvants and antigens with distinct physicochemical properties and are an excellent candidate platform for the development of multi-antigen vaccines.

## 1 Introduction

*Yersinia pestis*, the etiological agent of plague, is a gram-negative bacterium that is responsible for three pandemics that have shaped the course of human history and continues to be a concern for both biodefense and public health communities (1–3). Plague is a zoonotic disease, with major foci in Africa, Asia, and North and South America, leading to periodic human cases and outbreaks, particularly in Africa in recent years (4, 5). The most recent pandemic began in China, spread worldwide after reaching Hong Kong in 1894 and was considered by the World Health Organization to be resolved in 1960, by which time it had produced outbreaks on all continents with the exception of Antarctica (6, 7). Depending on the route of infection, plague can manifest as three major clinical forms: bubonic, septicemic, and pneumonic. Primary pneumonic plague, the result of direct inhalation of infectious aerosols, rapidly progresses to severe pneumonia following infection, with mortality rates approaching 100% in the absence of proper antibiotic treatment (8, 9). This route of transmission is of great concern since the window for antibiotic treatment is very narrow following symptom onset and late-stage pneumonic plague is transmissible person-to-person (8–12). Importantly, antimicrobial resistance has been documented in *Y. pestis*, including human clinical isolates from both bubonic and pneumonic plague patients (13–18). Currently, there is no licensed vaccine in the United States, and with the looming threat of increased antimicrobial resistance, a safe and effective *Y. pestis* vaccine is needed for protecting both the warfighter and the general population (19).

Significant effort has been directed toward developing subunit vaccines against *Y. pestis* due to their increased safety profile compared to live attenuated vaccines (LAVs) (20, 21). While subunit vaccines require adjuvants to elicit a robust and long-lasting immune response, they can be less effective than LAVs at inducing cellular immunity (22, 23), which is crucial for combating obligate intracellular pathogens (24, 25) and contributes to protection against facultative intracellular organisms including *Y. pestis* (26, 27). Despite being potentially more vulnerable to escape mutations in emerging pathogen variants, subunit vaccines do offer some advantages, such as reduced reactogenicity, consistency of formulation, and scalability of production. For instance, a recent safety study of a subunit plague vaccine with an aluminum adjuvant demonstrated high, persistent serological titers against one of the two primary antigens for up to 12 months, with no serious adverse events reported (28). Subunit plague vaccines in development have primarily targeted two proteins antigens: Fraction 1 (F1) and Low calcium response V (LcrV, or V) (29–39). The F1 protein, encoded by the *caf1* gene, is induced at 37°C to produce an anti-phagocytic capsule on the surface of *Y. pestis* (40, 41), while the V antigen is a multi-functional protein forms the tip of the type 3 secretion system injectisome (42–44), enabling the translocation of Yop effector proteins into host cells (45–47), and suppresses pro-inflammatory Th1 responses by stimulating IL-10 pathways (45, 48–50). A recombinant F1-V (F1V) fusion protein subunit vaccine has shown significant protective efficacy when formulated with Alhydrogel (Alu) and CpG2006 (35, 36, 39), but anti-F1V antibodies could also target non-native epitopes due to the chimeric nature of the fusion protein (39). Furthermore, this formulation is restricted to a specific antigenic variant of V, which can be polymorphic in some *Yersinia* species (51–53). Recent studies have demonstrated that the F1V vaccine can be combined with live attenuated vaccine strains in successful heterologous vaccination strategies against pneumonic plague (54–56).

To further pursue optimization of subunit vaccines for safety and efficacy, we explored a novel strategy combining F1 and V proteins with a self-assembling nanolipoprotein (NLP) complex. The NLPs, also known as nanodiscs or reconstituted high-density lipoproteins (rHDLs), take the form of discoidal bilayers that are stabilized by scaffold proteins based on the 22 kDa N-terminal fragment of apolipoprotein E4. When the bilayers are assembled with a bilayer forming lipid (DOPC) and a nickel-chelating lipid (DGS-NTA-Ni), the NLPs readily conjugate His-tagged proteins to the particle surface (57–60). With a particle diameter approximately 15 nm, this nanoparticle is a promising platform for subunit vaccines, with advantages including incorporation of proteins in their native form (including membrane proteins), variation and optimization of ratios between antigenic components, co-localized delivery of antigen and adjuvant, and the potential for combinatorial vaccines for two or more pathogens at once (57, 61, 62). Here, we used an established model of pneumonic plague, in which BALB/c mice were aerosol challenged with the virulent CO92 strain of *Y. pestis* after two doses of vaccine. Vaccine formulations including NLPs (F1:-, V:-, and F1:V:NLP) were compared for efficacy with an established vaccine containing F1V, an alum-based adjuvant, and the TLR9 agonist CpG ODN 2006 (35, 56). This study builds on previously generated data from other pathogens like influenza virus and *Bacillus anthracis*, which showed that incorporating NLPs in an antigen-adjuvant formulation can enhance immunity without compromising the safety profile (57, 63, 64).

## 2 Materials and methods

### 2.1 Bacterial culture and whole-cell antigen preparation

For use in immunological profiling, the wild-type *Y. pestis* CO92 strain (65) was grown on 5% sheep blood agar (SBA) plates for approximately 48 h at 28-30°C. Liquid culture of *Y. pestis* CO92 used as an antigen in immune assays was prepared in heart infusion broth (HIB) medium supplemented with 0.2% xylose (HIBX) and grown at 28-30°C for 21 h followed by switch to 37°C for an additional 3 h to upregulate the presentation of potential antigens. The bacteria were then harvested via centrifugation (10 minutes at 13,000 x g) and inactivated by exposure to approximately 21 kGy of gamma-radiation. This antigen preparation was designated CO92 TS (temperature shifted) (56). Bacteriological media were obtained from Thermo Fisher-Remel (Rockville, MD).

### 2.2 Preparation of vaccines for injection

NLPs were prepared as previously described (57–60). Briefly, lipids (DOPC and DGS-NTA-Ni) in chloroform were dried into a thin film using N_2_ and resuspended in PBS with 60 mM sodium cholate. After adding E4.22k scaffold protein (murine sequence), the assembly mixture was dialyzed overnight against PBS. Assembly mixtures were then purified by size exclusion chromatography (SEC), and fractions corresponding to pure NLPs were pooled and concentrated. NLPs were subsequently filter-sterilized, formulated with 0.1M trehalose, aliquoted, and lyophilized. To assemble NLP-based vaccines, lyophilized NLPs were rehydrated and mixed with His-tagged recombinant F1 or V.

Recombinant F1 and V proteins were expressed in cell-free reactions overnight at 25°C using the DASbox Mini Bioreaction System (Eppendorf, Hamburg, Germany) with proprietary *E. coli* lysates and proprietary culture media. F1 was expressed as a TwinStrep-TEV-F1-His construct and purified using three methods sequentially: 1) affinity chromatography using StrepTrap column with Tris buffers containing EDTA, TEV cleavage to remove the TwinStrep tag, 2) cation exchange using HiTrap Capto SP ImpRes using Tris buffers and a 1 M sodium chloride gradient, and 3) affinity chromatography using a HisTrap Excel column with Tris buffers containing the reducing agent TCEP. The expressed V protein contained only a histidine tag and utilized a two-step method: 1) affinity chromatography using a HisTrap Excel column with Tris buffers containing TCEP and 2) size-exclusion chromatography on a 26/60 Sephacryl column using an isocratic elution in 1X PBS. For both proteins, desired fractions from the final purification step were pooled, concentrated, then quantified using the BCA assay. Purity of the final products was assessed by SDS-PAGE (**Supplementary Figure 1**). Antigen endotoxin levels were assessed using limulus amebocyte lysate Endosafe-PTS cartridges from Charles River Laboratories (Wilmington, MA). Endotoxin levels for F1 and V were measured to be 13.7 EU/mg and 30.0 EU/mg, respectively. With antigen doses of 0.5 μg (see below), animals received 0.0068 EU/dose (F1), 0.015 EU/dose (V), or 0.022 EU/dose (F1 + V). These levels are significantly lower than cited thresholds of 0.1 EU/20 g mouse (www.fda.gov). All columns used for purification were purchased from Cytiva (Malborough, MA).

The *Y. pestis* F1 amino acid sequence (NCBI Accession WP_002216410.1) featured a C-terminal 6X His-tag and an R124N mutation to decrease cleavage during the self-assembly reaction. TEV cleavage is denoted by a period:

HMSAWSHPQFEKGGGSGGGSGGSAWSHPQFEKGSGENLYFQ.GMADLTASTTATATLVEPARITLTYKEGAPITIMDNGNIDTELLVGTLTLGGYKTGTTSTSVNFTDAAGDPMYLTFTSQDGNNHQFTTKVIGKDSRDFDISPKVNGENLVGDDVVLATGSQDFFVNSIGSKGGKLAAGKYTDAVTVTVSNQHHHHHH.

The *Y. pestis* LcrV amino acid sequence (NCBI Accession WP_002212981.1) featured a C-terminal 6X His-tag:

HMIRAYEQNPQHFIEDLEKVRVEQLTGHGSSVLEELVQLVKDKNIDISIKYDPRKDSEVFANRVITDDIELLKKILAYFLPEDAILKGGHYDNQLQNGIKRVKEFLESSPNTQWELRAFMAVMHFSLTADRIDDDILKVIVDSMNHHGDARSKLREELAELTAELKIYSVIQAEINKHLSSSGTINIHDKSINLMDKNLYGYTDEEIFKASAEYKILEKMPQTTIQVDGSEKKIVSIKDFLGSENKRTGALGNLKNSYSYNKDNNELSHFATTCSDKSRPLNDLVSQKTTQLSDITSRFNSAIEALNRFIQKYDSVMQRLLDDTSGKHHHHHH.

Conjugation of antigens to NLPs was verified by SEC using Cytiva Superose 6 Increase (5 x 150 mm) columns. SEC was used to verify that all His-tagged antigen was bound to the NLP at the chosen antigen:NLP ratios, with no unconjugated antigen observed. Dynamic Light Scattering was conducted in PBS using a Malvern Zetasizer Nano ZSP (Westborough, MA).

Recombinant F1V fusion protein was used as a positive control and to assess vaccine noninferiority. F1V was formulated under GMP regulations and stored at -80°C in single-use aliquots prior to use (66, 67).

Toll-like receptor 9 (TLR9) oligonucleotide (ODN) CpG ODN 2006 (CpG2006) was purchased from InvivoGen (San Diego, CA) and reconstituted in accordance with manufacturer recommendations. Alhydrogel (Alu) was sourced from InvivoGen.

Vaccines were formulated immediately prior to vaccination. For NLP formulations, lyophilized NLPs were rehydrated in water and mixed with F1 and/or V antigens (0.5 μg) at antigen-to-NLP ratios where no unconjugated antigens are observed. Formulations were then mixed with CpG and Alu such that the final formulations contained 10 μg of CpG and approximately 250 μg of Alu per dose.

### 2.3 Animals and vaccination studies

**Figure 1:**
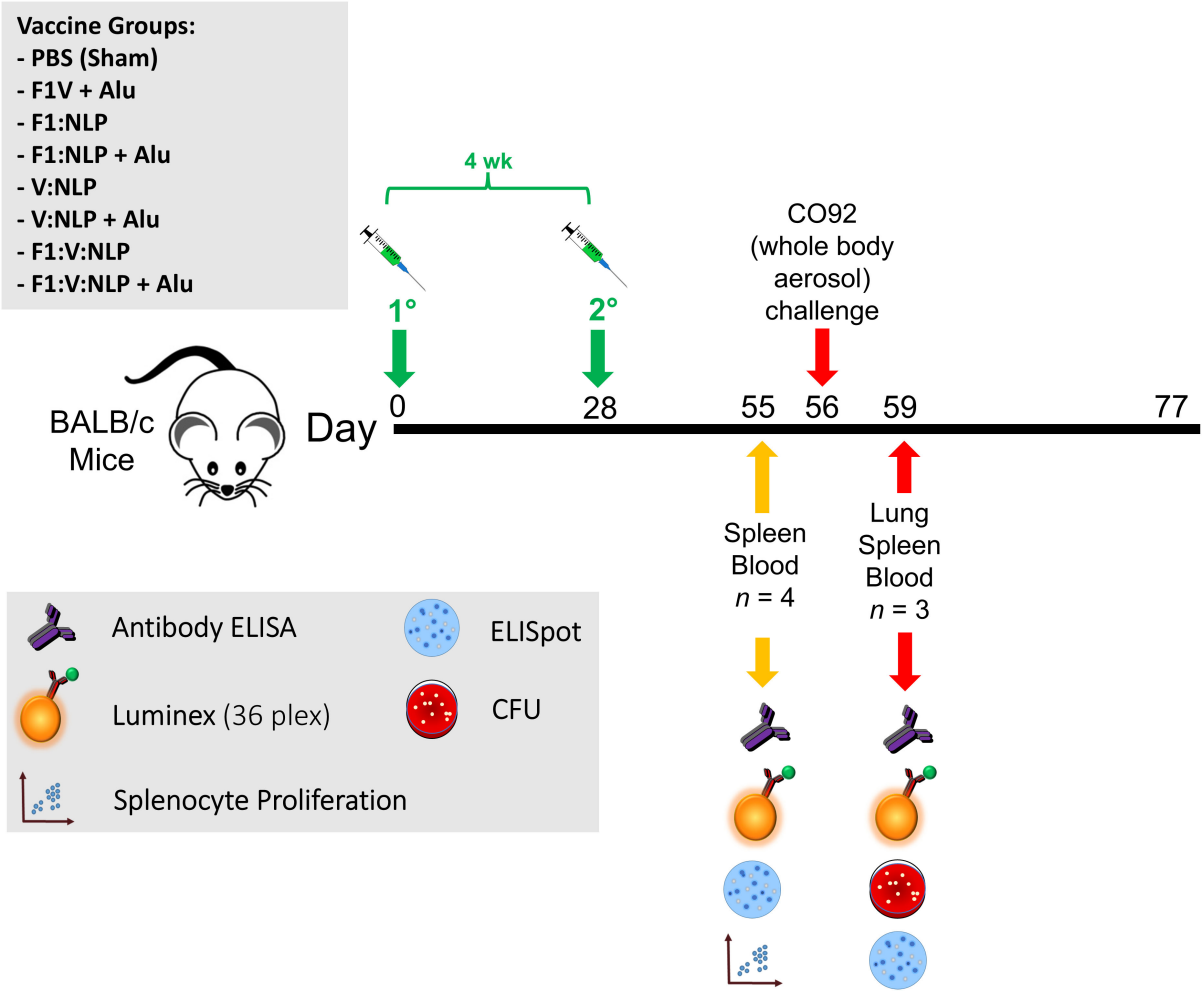
Overview of the immunization and challenge strategy for direct comparison of benchmark F1V vaccine and various NLP vaccine candidates that were challenged with *Y. pestis* CO92. The numbers with the degree sign (°) denote vaccine prime and boost.

Female BALB/c mice (*n* = 10 per experimental group) were obtained from Charles River (Frederick, MD) and were 7–9 weeks of age at time of vaccination. The vaccines described consisted of 0.5 μg of antigens (F1V, F1, or V), 250 μg of Alhydrogel and 5 μg of CpG. Mice were vaccinated twice subcutaneously, 21–28 days apart, and exposed four weeks later by the aerosol route to a lethal dose of *Y. pestis* CO92. Two independent studies were performed, with details shown in **Figure 1** and **Table 1**. In Experiments A and B, sera and spleens were collected from a cohort of mice (*n* = 4/group in A, *n* = 6/group in B) the day prior to challenge. In addition, in Experiment A lung, sera, and spleens were collected from a cohort of mice (*n* = 3/group) three days following challenge. The remaining mice (*n* = 9 or 10/group) were monitored for signs of infection for 21 days following challenge. Euthanasia of moribund animals was conducted in accordance with approved early endpoint intervention criteria.

**Table 1:** Experimental design.

Experiment	Vaccine formulation*	Route of administration	Vaccine prime	Vaccine boost	Aerosol challenge	Challenge dose
A	PBS (Sham)	subcutaneous	Day 0	Day 28	Day 56	5.3 x 10^5^ (8.0 LD_50_)
	F1V + Alu					
	F1:NLP					
	F1:NLP + Alu					
	V:NLP					
	V:NLP + Alu					
	F1:V:NLP					
	F1:V:NLP + Alu					
B	PBS (Sham)	subcutaneous	Day 0	Day 21	N/A	N/A
	F1V + Alu					
	F1:NLP					
	F1:NLP + Alu					
	V:NLP					
	V:NLP + Alu					
	F1:V:NLP					
	F1:V:NLP + Alu					

*0.5 µg F1V; 0.5 µg F1; 0.5 µg V; 250 µg Alhydrogel (Alu) and 5 µg CpG ODN 2006 in all cases.

### 2.4 Ethics statement

The animal research was conducted under an Institutional Animal Care and Use Committee (IACUC) approved protocol in compliance with the Animal Welfare Act, Public Health Service Policy on Humane Care and Use of Laboratory Animals, and other federal statutes and regulations relating to animals and experiments involving animals. USAMRIID is accredited by the AAALAC International and adheres to the principles stated in *The Guide for the Care and Use of Laboratory Animals* (National Research Council, 2011). Mice were checked daily for food and water and at least daily for assessment of clinical impact of the *Y. pestis* infection. Whenever possible, euthanasia of moribund animals was conducted in accordance with approved early endpoint intervention criteria. Mice were evaluated daily after exposure to aerosolized *Y. pestis*; scores of 0–2 represented normal mice, scores of 3–7 indicated significant clinical manifestations and these mice warranted multiple clinical assessments per day, and final scores of 8 or greater indicated severe clinical manifestations and mice were euthanized immediately. When mice met pre-determined euthanasia criteria and they were not in the sampling cohorts, mice were euthanized by CO_2_ exposure (flow rate 6–11 ft^3^/h) or by barbiturate overdose through intraperitoneal injection (approximately 0.15 mL for 20 g of body weight) of Euthasol^®^ euthanasia solution (or equivalent) and then death was confirmed by cervical dislocation.

### 2.5 Exposure of vaccinated mice to virulent *Y. pestis* CO92

Mice were exposed to aerosolized challenge doses of virulent *Y. pestis* CO92 strain. Bacterial preparation for aerosol challenge involved growing bacteria on tryptose blood agar (TBA) base slants for approximately 48 h at 28-30°C. Bacteria from TBA slants were then suspended in HIBX to an initial OD_620_ of approximately 0.01 and incubated for approximately 24 h at 28–30°C. Then, the cultures were harvested by centrifugation (10 min at 13,000 x g) and suspended in HIB medium (no xylose) to the concentration yielding the number of LD_50_ doses indicated in the text and figure legend. A solution of 10 mM potassium phosphate, pH 7.3–7.4 (KPhos) was used to dilute bacterial inocula. Challenge doses were determined by serial dilutions in KPhos buffer and plating on SBA.

Mice were transferred to wire mesh cages and were placed in a whole-body aerosol chamber within a class three biological safety cabinet located inside a BSL-3 laboratory. Mice were exposed to aerosols of *Y. pestis* CO92 created by a three-jet collison nebulizer. The LD_50_ of *Y. pestis* CO92 in BALB/c mice is approximately 6.8x10^4^ inhaled CFU. Samples were collected from the all-glass impinger (AGI) vessel and analyzed by performing CFU calculations to determine the inhaled dose of *Y. pestis* (68, 69). The actual inhaled dose was calculated to be approximately 5.3x10^5^ CFU (approximately 8.0 LD_50_s).

### 2.6 Determination of bacterial burden

The tissues collected from necropsied mice, 3 days following challenge, included lung, spleen, and blood. For sample collections, mice were deeply anesthetized with approximately 0.3 mL/20 g of body weight with a mixture of ketamine (10 mg/mL)-acepromazine (1 mg/mL)-xylazine (2 mg/mL), underwent a terminal blood collection via the axillary vessels, and then were euthanized by cervical dislocation prior to organ harvesting. The organs were weighed, homogenized in 1 mL KPhos per sample with disposable PRECISION™ homogenizers (Covidien, Dublin, Ireland), and the CFU in the homogenate were quantified by plating on SBA plates. Undiluted homogenate and ten-fold dilutions in KPhos were plated in duplicate. The limit of detection (LOD) was approximately 100 CFU/mL blood or 5 CFU/organ. After CFU determinations, samples were radiation-inactivated, sterility checked and stored at -80°C for immunological analyses.

### 2.7 Evaluation of humoral immune responses

Sera from 27 days post vaccine boost and 3 days post-challenge blood collection were measured for Immunoglobulin G (total IgG along with IgG1 and IgG2a subclasses) antibody levels by semi-quantitative endpoint ELISA in 96-well Immulon 2HB plates (Thermo Fisher, Rochester, NY). Plates were incubated overnight with antigens at 4°C to coat wells. Coating antigen solutions were either pure proteins F1V, F1 or V at 2 µg/mL, or inactivated *Y. pestis* CO92 TS at 10 µg/mL in 0.1 M carbonate buffer, pH 9.5. Wells were washed five times with 1X PBS including 0.05% Tween 20. Two-fold dilutions of serum in PBS/0.05% Tween 20 were made in triplicate and incubated for 60 min at 37°C, then washed five times, blocked with 1% Casein in PBS (Pierce) for 30 min at 37°C, and signal detected as previously described (35). Detection antibodies were horseradish peroxidase-conjugated goat anti-IgG, -IgG1, or -IgG2a (Southern Biotechnology) at dilution of 1:5,000. After 30 min at 37°C with detection antibodies, plates were washed five times, incubated with 3,3’,5,5’-tetramethylbenzidine substrate (Pierce) for 20 min at 37°C, and the reaction stopped with 2 N sulfuric acid.

A Biotek ELx808 plate reader was used to quantify the amount of bound antibody by colorimetric measurement at 450 nm. Results are reported as the geometric mean (GM) and geometric standard error (GSE) of the reciprocal of the highest dilution giving a mean OD of at least 0.1 ± 1 SD at 450 nm (570 nm used as reference wavelength), then triplicates averaged. The limit of detection was 50, with values ≤ 50 considered negative. To assess the avidity of serum antibodies, we performed the ELISA as described above, in parallel conditions with or without an additional 15 min incubation in 6 M urea at room temperature, immediately after incubation with samples. We chose the lowest dilution that had an OD_450_ below 1.5 and calculated the avidity index based on the values at this dilution, as (OD with urea/OD with wash buffer) x 100%.

### 2.8 Evaluation of cellular immune responses by ELISpot

Groups of mice were euthanized 27 days after the vaccine boost, and splenocytes were isolated using minimal disruption as described previously (54). Briefly, spleens were excised from mice (*n* = 4 [Experiment A] and *n* = 6 [Experiment B] mice per group) and disaggregated in RPMI 1640 medium (Gibco, Grand Island, NY). Red blood cells in the splenocyte preparation were lysed with Ammonium-Chloride-Potassium (ACK) Lysing Buffer (Gibco) the extract was then diluted with RPMI 1640 medium and cells pelleted by centrifugation at 335 x g for 10 min. Splenocytes were resuspended in CTL-Test medium (ImmunoSpot, Shaker Heights, OH) and counted using a TC20 Cell Counter (Bio-Rad, Hercules, CA), then all samples were diluted in RPMI complete medium to normalize cell concentrations. Complete medium was RPMI-1640 including 10% fetal bovine serum (HyClone, Logan, UT), 100 U/ml penicillin-streptomycin (Gibco), 1x MEM Non-essential Amino Acid Solution (Sigma, St. Louis, MO), 1 mM sodium pyruvate (Sigma), and 55 µM β-mercaptoethanol (Gibco). ELISpot kits from ImmunoSpot were used (Mouse IgG1/IgG2a Double-Color ELISPOT Assay) and manufacturer’s instructions followed, as described here in brief.

For B cell ELISpot, splenocytes were first stimulated for 6 days with 1x B-Poly S Polyclonal B cell Stimulator (ImmunoSpot) in complete medium. Stimulation took place at 37°C/5% CO_2_, with 5x10^6^ cells/mL in 48-well tissue culture plates at a total volume of 1.6 mL for each sample. Plates provided with the kit were coated with F1V (10 µg/mL), V (10 µg/mL), or F1 (10 µg/mL) protein or positive control wells coated with Anti-Igκ and Anti-Igλ Capture Ab (ImmunoSpot). Antigen-treated plates were washed, splenocytes were added at 5x10^5^ cells/well and incubated for approximately 8 h, then washed, developed and spots detected as per manufacturer’s instructions. Spot forming units (SFU), indicative of antigen specific antibody secreting cells, were normalized to the 1,000 SFU based on the number of total antibody secreting B cells. All ELISpot assays were performed with each sample in two duplicate wells.

### 2.9 Cytokine quantification by Luminex

For cytokine determination in vaccinated mice pre-challenge, splenocytes were isolated and counted 27 days after the vaccine boost. Cells were incubated in RPMI complete medium with antigens F1V (25 µg/mL), F1 (25 µg/mL), or V (25 µg/mL) at 37°C/5% CO_2_. Splenocytes stimulated with medium only were included as negative controls, while positive controls were stimulated with 100 ng/mL phorbol 12-myristate 13-acetate (PMA) and 500 ng/mL ionomycin. After approximately 48 h incubation, plates were centrifuged at 1,200 x g for 10 min, then supernatants were harvested for evaluation of cytokine expression, as described below.

For mice post-challenge, lungs and spleens were homogenized at 3 days post-infection as described in section 2.6; homogenates were frozen at -80°C and irradiated prior to quantification of cytokines and other proteins in the tissue. All samples were thawed from cryopreservation, then centrifuged at 10,000 x g for 10 min to minimize debris.

Supernatants and homogenates were measured for cytokine expression levels using the Cytokine & Chemokine 36-Plex Mouse ProcartaPlex panel (Thermo Fisher) and MagPix instrument per manufacturer’s instructions. Cytokine results that were above or below the highest or lowest values of the standard curve were imputed to the value of the highest or lowest standard. In addition, any cytokine whose standard curve had low R^2^ value (< 0.95) or a bead count of < 35 was removed from analysis. Cytokine levels are expressed as the fold change relative to PBS and in pg/mL.

### 2.10 Flow cytometry

Splenocytes from mice 27 days post-vaccine boost were isolated and cryopreserved in freezing medium, then transferred to liquid nitrogen vapor storage. After thawing and counting, cells were labeled with viability dye (LIVE/DEAD Fixable Green or Violet, Thermo Fisher), washed with FACS buffer, incubated with 1:200 dilution of Mouse Fc Block (5534142, BD), then stained with fluorescently conjugated antibodies and fixed in 2% formaldehyde (Thermo Fisher, Rockford, IL). The surface markers used for defining subsets were CD3e (antibody clone 145-2C11), CD19 (clone 1D3), CD4 (clone GK1.5), CD8a (clone 53-6.7), CD62L (clone MEL-14), CD11c (clone N418), CD11b (clone M1/70), CD40 (clone 3/23), and CD80 (clone 16-10A1). FACS buffer was 1% bovine serum albumin (SH30574, HyClone) in PBS, and freezing medium was 10% DMSO (D2660, Sigma) and 90% fetal bovine serum (SH30396, HyClone). Samples were run on a FACSCanto II (BD) and analyzed in FlowJo v10.8.

### 2.11 Statistical analyses

The mouse survival rates at selected time points were compared by Fisher exact test and the time to death or euthanasia (TTD) were analyzed by Log-rank test for the comparison against PBS group. For ELISA, pairwise treatment groups were compared by negative binomial generalized linear mixed model. Negative binomial regression was used for the flow cytometry data analysis. Data (ELISpot and Luminex) were log10 transformed prior to analysis. For ELISpot and Luminex cytokine results, pairwise treatment groups were compared by linear mixed effects model. The multiplicity was adjusted by Tukey’s method. Analysis was implemented in SAS version 9.4 (SAS Institute Inc., Cary, NC).

## 3 Results

### 3.1 NLPs can be readily formulated with F1 and V antigens

**Figure 2:**
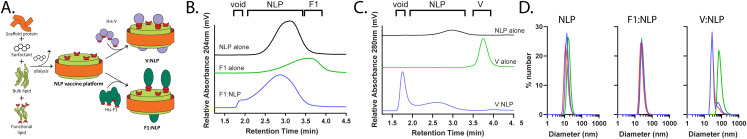
Development and characterization of NLP-based vaccine. **(A)** Schematic of NLP assembly and conjugation of His-tagged *Y. pestis* antigens. **(B, C)** Representative SEC chromatograms of F1:NLP **(B)** and V:NLP **(C)** conjugates, correlating NLP alone (black traces), antigen alone (green traces), and antigen:NLP conjugates (blue traces). Relative retention time regions corresponding to void volume (void), NLP and antigen:NLP (NLP), and unconjugated antigen (F1 or V) are indicated. **(D)** DLS histograms of NLP alone, F1:NLP and V:NLP formulations (number mean, triplicate measurements). The red, green, and blue histograms represent the replicate measurements for each sample.

NLPs were prepared through a well-established self-assembly reaction. Briefly, recombinantly expressed apolipoprotein scaffold (22 kDa N-terminal domain of ApoE) is solubilized in a surfactant solution with both bilayer-forming lipids (DOPC) and a lipid with a nitrilotriacetic acid (NTA) modified head group chelating nickel. As the surfactant is removed via dialysis, the components spontaneously self-assemble into NLPs. The inclusion of the nickel lipid creates functionalized NLPs that are capable of binding histidine-tagged proteins (**Figure 2A**). Conjugation of his-tagged proteins to the NLPs can give varied results in terms of ideal protein:NLP molar ratios, total protein binding capacity, and the size of the particles after conjugation (with some proteins tending to induce the formation of large aggregates); therefore, conjugation of the F1 and V antigens to the NLP was assessed separately using size exclusion chromatography (SEC) and dynamic light scattering (DLS). Screens were performed in the absence of the adjuvants used in the final vaccine formulations.

NLPs elute as a single peak with a retention time (t_r_) of 3 min, providing a baseline for comparison of NLPs with protein conjugates. Larger species, such as protein:NLP conjugates, will elute earlier (i.e., closer to the column void volume at 2 min) and any smaller species, such as monomeric unconjugated protein, will elute after the NLP (~ 4 min). Initial conjugation screens were conducted at increasing antigen:NLP ratios, ranging from 0:1 to 16:1 (data not shown). SEC was used to identify conjugation ratios that showed complete antigen binding (e.g., no unconjugated antigen) while minimizing larger species (i.e., elution in the void volume). These screens identified that F1 and V ratios per NLP of 4:1 and 8:1 (respectively) demonstrated successful conjugation to the NLP (**Figures 2B, C**). For the F1:NLP ratio of 4:1, addition of the protein to the NLP causes a shift to shorter retention times, indicating the successful conjugation and formation of slightly larger F1:NLP particles, without any indication of free (or unconjugated) F1 protein (**Figure 2B**). Conjugation of V to the NLP at an 8:1 ratio was also successful, with all V protein conjugated to the NLPs. Some larger multimeric NLP species with V are observed (~2 min), suggesting some degree of interparticle multimerization (**Figure 2C**).

The formulations were further characterized by DLS to evaluate average size of the NLPs and the NLP conjugates (**Figure 2D**). The diameter of the particles, based on number mean, was analyzed for the NLP alone and the F1:NLP at a 4:1 ratio and V:NLP at an 8:1 ratio. The average size of the NLPs without conjugated protein was 15.1 ± 1.7 nm. The addition of F1 protein resulted in an increase in average particle size to 26.8 ± 1.7 nm. The addition of V resulted in a mix of larger NLP particles (~37 nm) and some multimeric species (> 85nm), with an average size of 53.4 ± 28.6 nm. These results mirror the trends seen by SEC, and together demonstrate that the NLPs can be readily formulated with the F1 and V antigens at tailored ratios for use in downstream vaccine studies.

### 3.2 The cytokine response generated with the NLP platform is comparable to that of the traditional subunit F1V vaccine when Alhydrogel is included

To date, F1V admixed with the adjuvants Alu and CpG2006 remains the benchmark subunit vaccine for *Y. pestis* in our laboratory (35, 36, 39, 70). Unfortunately, the chimeric nature of the fusion protein may potentially induce immune responses to novel linear or conformational epitopes that are absent from native protein structures and hence diminish the protective efficacy of the vaccine. To lessen this possibility, we evaluated individual F1 and/or V antigens conjugated to the Ni-chelating NLP-based vaccine delivery platform and compared it to the F1V formulation. Mice were vaccinated with two doses of the vaccines, four weeks apart and the cytokine response was assessed in splenocytes approximately four weeks after the last vaccine dose, against F1V, V, and F1 proteins (**Table 2** and **Supplementary Table 1**). As expected, the benchmark F1V vaccine induced a strong anti-F1V, V, and F1 cytokine response, based on the fold change differences relative to sham (PBS) vaccinated mice. NLP formulations, much like F1V vaccine, required addition of Alhydrogel (Alu) to induce a similar cytokine response. F1:V:NLP splenocytes had little cytokine response upon restimulation, but F1:V:NLP + Alu splenocytes produced a strong response, with many cytokines upregulated at similar or higher levels than the results from the benchmark F1V vaccine. For both vaccines, the pattern of which cytokines were most induced in F1V-restimulated splenocytes was very similar, with exceptions being IL-17A and Eotaxin which were significantly upregulated in the F1V group but not F1:V:NLP + Alu.

Results from restimulation with individual V and F1 proteins were especially encouraging (**Tables 2B, C**). Splenocytes from F1:V:NLP + Alu vaccinated mice secreted more than twice the IL-5, IL-13, IL-2, IFN-γ, IL-4, IL-3, GM-CSF, IL-22, IL-10, and LIF than splenocytes from F1V vaccinated mice when restimulated with F1 alone. Similar results were seen for restimulation with V alone, although less pronounced because splenocytes from F1V vaccinated mice already had a stronger response to V than F1 restimulation. These data suggest that the NLP formulation with V and F1 included as separate components is more effective than F1V fusion protein at presenting these two antigens to the immune system. As would be expected, the cytokine response of the NLP formulations was antigen-specific, with a very limited recall response induced by V restimulation of splenocytes from F1 vaccinated mice or vice versa.

**Table 2:** The fold change relative to PBS group in cytokine responses obtained 27 days after vaccination and stimulation of splenocytes with (A) F1V, (B) V and (C) F1 antigens.

A							
F1V							
Cytokine^a^	F1V + Alu	F1:NLP	F1:NLP + Alu	V:NLP	V:NLP + Alu	F1:V:NLP	F1:V:NLP + Alu
IL-5	**374.70**	1.03	**671.68**	1.23	**2,194.98**	2.13	**1,254.75**
IL-13	**99.49**	1.00	**242.00**	1.27	**453.72**	1.96	**309.22**
IL-2	**73.44**	3.82	**66.42**	2.51	**107.54**	**6.25**	**70.95**
IFN gamma	**41.98**	3.56	**80.31**	2.32	**175.53**	5.91	**81.42**
IL-6	**38.59**	2.72	**63.80**	3.62	**42.56**	7.55	**46.40**
IL-4	**36.36**	0.87	**24.42**	1.11	**69.72**	3.53	**55.39**
IL-3	**35.12**	1.94	**41.56**	1.41	**46.54**	5.91	**43.33**
GM-CSF	**31.50**	1.00	**41.52**	1.25	**47.04**	2.00	**37.71**
IL-17A (CTLA-8)	**24.19**	1.13	4.99	1.66	4.96	2.47	3.87
IL-22	**14.61**	1.00	**16.36**	1.01	**12.29**	1.86	**12.23**
TNF alpha	**12.34**	0.79	**11.31**	0.95	**18.85**	0.91	**12.55**
MIP-1 alpha (CCL3)	**11.65**	1.10	**17.55**	1.35	**19.45**	1.89	**21.03**
MCP-3 (CCL7)	**8.03**	1.58	**9.17**	1.39	**9.49**	3.86	**9.60**
IL-10	**7.73**	1.00	**5.27**	1.23	**28.94**	1.66	**15.63**
Eotaxin	**6.43**	1.00	1.59	1.00	1.30	1.07	2.02
IP-10 (CXCL10)	6.18	1.61	7.91	1.24	5.86	4.79	7.18
MIP-1 beta (CCL4)	5.04	1.10	**7.78**	1.31	**8.80**	1.58	**10.15**
MIP-2 alpha (CXCL2)	**4.45**	1.28	**4.91**	1.15	**4.96**	1.45	**4.74**
LIF	3.89	0.98	1.49	0.98	1.02	1.00	2.54
IL-1 beta	2.97	1.26	3.63	1.34	**4.54**	1.44	3.62
IL-12p70	2.76	0.96	2.10	0.62	**4.45**	0.99	3.05
IL-15	2.37	1.00	1.81	1.00	**3.03**	0.96	**2.75**
IL-27	2.26	1.00	1.09	1.00	1.13	1.00	1.87
RANTES (CCL5)	1.44	0.87	1.72	1.01	1.62	1.07	1.55
GRO-alpha (CXCL1)	1.35	1.09	1.20	1.02	0.74	1.25	1.28
IL-9	1.21	1.00	1.00	1.00	1.14	1.00	1.25
IFN alpha	1.03	1.00	1.00	1.00	1.00	1.00	0.95
ENA-78 (CXCL5)	1.02	1.00	1.00	1.00	1.00	1.00	0.90
G-CSF	1.00	1.00	1.00	1.00	1.00	1.00	0.95
IL-18	1.00	1.00	1.30	1.00	2.16	1.00	1.80
IL-31	1.00	1.00	1.00	1.00	1.00	1.00	0.95
IL-23	1.00	1.00	1.00	1.00	1.00	1.00	0.95
IL-28	0.93	1.00	1.00	1.00	1.00	1.00	0.93
IL-1 alpha	0.88	1.00	1.00	1.00	1.00	1.00	0.77
M-CSF	**0.64**	1.00	0.92	1.00	1.00	1.00	0.75
B							
V							
Cytokine^a^	F1V + Alu	F1:NLP	F1:NLP + Alu	V:NLP	V:NLP + Alu	F1:V:NLP	F1:V:NLP + Alu
IL-5	**217.70**	1.00	**24.65**	2.24	**3,394.56**	2.06	**1,070.63**
IL-13	**49.86**	1.00	**15.65**	1.84	**598.55**	2.33	**267.90**
IL-4	**32.97**	1.29	4.91	2.92	**147.14**	4.44	**74.32**
IL-6	**25.72**	0.56	1.89	5.19	**52.44**	4.18	**31.37**
GM-CSF	**16.05**	1.09	2.44	2.13	**74.36**	2.78	**23.80**
IL-2	**15.58**	1.30	4.81	2.97	**85.80**	2.45	**22.61**
IL-3	**13.75**	1.09	3.64	1.69	**43.96**	2.62	**19.41**
IL-17A (CTLA-8)	8.42	0.88	1.48	2.70	**21.37**	4.47	6.94
IL-22	7.12	0.95	2.60	2.51	**35.92**	3.69	**15.60**
IFN gamma	6.68	0.45	1.59	2.40	**94.83**	2.20	18.91
Eotaxin	**4.96**	1.00	1.07	1.23	1.99	1.07	1.76
IL-10	**4.94**	1.00	1.07	1.94	**33.06**	2.16	**14.95**
MCP-3 (CCL7)	4.42	1.06	1.85	1.67	**5.80**	1.80	4.25
MIP-1 alpha (CCL3)	3.72	0.77	1.79	1.39	**13.81**	1.12	**6.60**
IL-1 beta	3.18	0.80	1.83	2.24	**7.10**	1.54	**4.29**
TNF alpha	3.02	0.61	1.34	1.29	**7.95**	0.49	3.83
IP-10 (CXCL10)	2.34	0.90	1.22	1.74	4.14	1.58	2.53
MIP-1 beta (CCL4)	1.92	0.88	1.34	1.26	**8.92**	1.14	4.78
IL-12p70	1.48	0.87	0.74	0.90	**4.76**	1.18	2.12
LIF	1.39	1.00	1.00	1.14	3.77	0.97	2.27
IL-15	1.23	0.85	0.78	1.14	**3.41**	0.86	2.47
IL-27	1.20	1.00	1.00	1.26	2.23	1.14	1.71
MIP-2 alpha (CXCL2)	1.10	0.72	1.03	0.94	1.67	0.66	1.14
GRO-alpha (CXCL1)	1.06	0.78	0.99	1.15	0.85	0.87	0.84
IL-9	1.00	1.00	1.00	0.99	**2.36**	1.00	1.51
G-CSF	1.00	1.00	1.00	1.00	1.00	1.00	0.97
IL-31	1.00	1.00	1.00	1.00	1.00	1.00	0.97
IL-18	1.00	1.00	1.00	1.00	**3.72**	1.00	1.77
IL-1 alpha	1.00	1.00	1.00	1.00	1.00	1.00	0.82
IL-28	1.00	1.00	1.00	1.00	1.00	1.00	0.97
IL-23	1.00	1.00	1.00	1.00	1.00	1.00	0.97
IFN alpha	0.96	1.00	1.00	1.00	1.06	1.00	0.97
ENA-78 (CXCL5)	0.88	1.00	1.00	1.00	1.00	0.96	**0.79**
RANTES (CCL5)	0.87	0.77	1.16	0.94	1.30	0.95	1.00
M-CSF	0.76	1.00	1.00	1.00	0.91	1.00	0.87
C							
F1							
Cytokine^a^	F1V + Alu	F1:NLP	F1:NLP + Alu	V:NLP	V:NLP + Alu	F1:V:NLP	F1:V:NLP + Alu
IL-5	**51.18**	4.98	**2,346.70**	0.89	2.13	5.39	**1,107.76**
IL-17A (CTLA-8)	**21.02**	**11.46**	**128.09**	1.66	1.21	**12.57**	**26.74**
IL-13	**20.42**	4.14	**410.55**	0.84	2.85	4.11	**233.80**
IL-4	**20.23**	3.17	**65.10**	0.97	2.64	4.82	**54.85**
IL-6	**17.18**	5.46	**72.93**	0.96	1.54	3.98	**35.64**
IL-2	**15.67**	**6.90**	**128.05**	1.08	2.25	**6.36**	**37.46**
IL-3	**14.61**	4.65	**93.87**	0.57	1.32	**7.27**	**42.50**
GM-CSF	**9.02**	2.16	**67.95**	1.13	1.09	2.89	**35.04**
IL-22	5.33	5.38	**63.51**	1.43	1.23	5.31	**28.07**
Eotaxin	**5.17**	0.99	2.21	0.99	1.00	1.15	1.93
IL-10	3.21	1.93	**17.49**	1.00	1.02	2.71	**11.30**
MCP-3 (CCL7)	2.73	1.82	3.21	1.06	1.24	1.93	2.79
IL-27	2.22	0.65	**6.79**	0.74	0.76	1.73	**4.26**
MIP-1 alpha (CCL3)	1.57	1.09	**7.53**	0.88	1.06	1.10	3.23
IL-1 beta	1.47	1.41	3.04	1.21	1.57	1.58	2.21
IFN gamma	1.40	2.71	30.21	0.21	0.94	1.46	13.95
TNF alpha	1.40	1.09	**3.93**	1.00	1.22	0.90	2.40
LIF	1.36	1.00	**9.73**	1.00	1.00	1.03	**4.09**
MIP-1 beta (CCL4)	1.32	1.10	**9.23**	1.02	1.04	1.30	4.28
IL-12p70	1.30	0.56	4.26	0.82	0.64	1.32	2.99
IP-10 (CXCL10)	1.24	2.16	3.49	0.68	0.73	1.63	2.18
IFN alpha	**1.18**	1.00	1.00	1.00	1.00	1.00	0.91
IL-9	1.12	1.00	**7.52**	1.00	1.00	1.05	**3.57**
ENA-78 (CXCL5)	1.09	1.00	1.00	1.00	1.00	1.00	0.97
IL-28	1.00	1.00	1.00	1.00	1.00	1.00	**0.76**
IL-31	1.00	1.00	1.00	1.00	1.00	1.00	**0.90**
G-CSF	1.00	1.00	**0.83**	1.00	1.00	1.00	0.86
IL-23	1.00	1.00	1.00	1.00	1.00	1.00	**0.90**
MIP-2 alpha (CXCL2)	0.99	0.95	2.54	1.10	0.93	0.77	1.49
GRO-alpha (CXCL1)	0.92	1.02	1.29	0.90	0.68	0.90	0.98
IL-15	0.89	0.81	**3.92**	0.68	0.57	1.40	2.30
RANTES (CCL5)	0.82	0.92	1.39	0.90	0.93	0.96	0.98
IL-1 alpha	0.78	1.00	0.96	1.00	1.00	1.00	0.94
IL-18	0.60	0.60	2.39	0.60	0.60	0.60	2.88
M-CSF	**0.51**	1.00	0.93	1.00	1.00	1.00	0.71

a The cytokine results are shown as the ratio to PBS (Sham), and are based on the Geometric Mean (pg/mL). Bolded (*p* < 0.05).

Vaccines were given 28 days apart. (data from experiment A, *n* = 4). 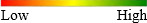

A similar cytokine profile was recapitulated in mice that received prime and boost doses 21 days apart rather than 28 (**Table 3**, **Supplementary Table 2**). Again, mice receiving NLP vaccine formulations had a strong splenocyte cytokine response against the *Y. pestis* antigen(s) in the vaccine, with the strength of this response dependent on the presence of Alu. Several cytokines that were not significantly induced above the sham (PBS) group in Experiment A were seen here to be significantly induced in F1V or F1:V:NLP + Alu groups, including IL-27, GRO-α and G-CSF (induced in F1:V:NLP + Alu) and IL-12p70, IL-18, and LIF (upregulated in both). In addition, unlike Experiment A, Eotaxin did not have enhanced production in the F1V group, and IL-17A was significantly induced in both groups rather than only in F1V. However, the apparently increased sensitivity of this experiment went along with more false positives in which splenocytes from mice vaccinated with F1 alone were restimulated by V antigen, or vice versa.

**Table 3:** The fold change relative to PBS group in cytokine responses obtained 27 days after vaccination and stimulation of splenocytes with (A) F1V, (B) V and (C) F1 antigens.

A							
F1V							
Cytokine^a^	F1V + Alu	F1:NLP	F1:NLP + Alu	V:NLP	V:NLP + Alu	F1:V:NLP	F1:V:NLP + Alu
IL-5	**1,509.26**	1.46	**229.49**	2.32	**2,346.74**	**6.89**	**1,873.20**
IL-13	**474.63**	2.59	**95.42**	2.93	**589.79**	**12.76**	**490.06**
IL-3	**228.50**	**4.63**	**34.19**	3.18	**237.46**	**17.52**	**186.67**
IL-17A (CTLA-8)	**228.22**	4.19	**9.07**	**7.44**	**131.10**	**69.40**	**118.75**
IL-4	**94.10**	**3.59**	**17.80**	**4.94**	**167.66**	**12.90**	**117.49**
IL-2	**89.55**	**3.72**	**20.59**	1.75	**68.05**	**7.22**	**43.75**
IFN gamma	**79.04**	5.65	**12.83**	4.18	**149.80**	**35.95**	**95.09**
IL-22	**55.05**	**5.74**	**10.91**	3.10	**88.93**	**35.87**	**77.42**
GM-CSF	**42.03**	2.25	**7.50**	2.21	**41.84**	**7.06**	**33.53**
IL-18	**21.70**	3.81	**7.04**	2.78	**29.40**	**14.86**	**26.39**
LIF	**19.80**	1.75	2.61	2.28	**31.74**	**6.60**	**25.14**
IL-6	**15.20**	1.87	**5.70**	2.34	**26.32**	**11.42**	**35.07**
IL-12p70	**7.36**	1.50	2.74	1.68	**9.46**	**4.84**	**8.49**
IL-10	**6.22**	1.09	1.53	1.19	**16.26**	**3.31**	**13.74**
IP-10 (CXCL10)	**4.92**	1.39	1.54	1.01	**11.08**	**9.17**	**13.06**
IL-15	**4.42**	2.11	2.50	1.87	**2.79**	**4.00**	2.32
MIP-1 alpha (CCL3)	**3.76**	1.36	**1.82**	1.74	**4.59**	**2.99**	**4.10**
MIP-1 beta (CCL4)	**3.12**	1.34	1.55	1.55	**5.52**	**3.36**	**4.51**
TNF alpha	**2.97**	1.33	1.56	1.36	**4.07**	**2.47**	**3.85**
IL-23	**2.91**	0.79	0.92	**0.45**	**2.36**	0.96	2.03
M-CSF	**2.61**	0.75	0.82	0.66	**2.14**	1.07	1.68
ENA-78 (CXCL5)	**2.57**	1.10	1.09	1.43	**2.57**	2.50	**3.55**
IL-9	**2.47**	1.01	1.15	1.00	**2.69**	1.09	**1.71**
IL-1 alpha	**2.34**	0.57	0.92	0.82	2.24	0.98	1.86
IL-27	**2.25**	0.97	1.18	1.29	**4.07**	2.14	**3.08**
MCP-3 (CCL7)	2.04	1.06	1.52	1.10	**5.19**	**7.34**	**12.39**
G-CSF	1.97	1.08	2.11	1.69	**2.90**	**2.47**	**3.14**
GRO-alpha (CXCL1)	1.91	1.33	0.60	0.81	**4.90**	**6.21**	**8.46**
IL-1 beta	1.67	1.03	1.26	1.48	**2.64**	1.87	**2.75**
MCP-1 (CCL2)	1.54	0.97	1.31	1.06	**4.21**	**7.44**	**9.60**
Eotaxin	1.20	1.04	1.05	0.96	**1.23**	1.14	**1.43**
RANTES (CCL5)	1.14	1.02	1.00	1.08	**1.47**	**1.50**	1.45
IL-31	0.93	1.04	1.16	1.00	0.85	1.11	0.71
IL-28	0.89	1.04	1.19	0.96	0.91	1.17	0.84
B							
V							
Cytokine^a^	F1V + Alu	F1:NLP	F1:NLP + Alu	V:NLP	V:NLP + Alu	F1:V:NLP	F1:V:NLP + Alu
IL-5	**768.03**	2.17	2.62	1.67	**1,081.83**	**5.79**	**1,294.69**
IL-13	**216.03**	2.53	3.15	1.98	**247.67**	**8.67**	**273.40**
IL-3	**105.73**	**4.48**	2.80	3.50	**134.53**	**12.47**	**105.80**
IL-17A (CTLA-8)	**83.07**	**8.38**	**4.74**	**14.23**	**90.61**	**77.12**	**111.66**
IL-2	**40.28**	**2.51**	2.00	2.08	**33.08**	**4.68**	**22.23**
IL-4	**38.10**	**3.18**	**4.75**	2.86	**80.64**	**7.45**	**53.82**
IL-22	**25.24**	**5.14**	1.99	3.81	**40.30**	**19.42**	**39.82**
IFN gamma	**21.77**	5.46	2.65	1.86	**76.68**	**15.68**	**45.84**
GM-CSF	**15.58**	1.93	1.27	1.21	**21.63**	**4.19**	**15.95**
IL-18	**10.57**	3.94	2.67	1.75	**17.45**	**8.66**	**15.66**
IL-6	**10.25**	1.66	**4.70**	2.02	**19.44**	**10.16**	**25.40**
LIF	**9.92**	1.42	0.85	1.62	**19.79**	**5.08**	**13.51**
IL-15	**5.49**	**3.88**	2.39	1.90	2.62	**5.30**	2.50
IL-12p70	**4.24**	1.64	1.37	1.13	**6.53**	**3.32**	**5.84**
IP-10 (CXCL10)	3.49	1.10	1.30	0.87	**8.52**	**7.46**	**8.88**
IL-10	**3.26**	1.11	0.93	1.13	**10.11**	**2.64**	**8.58**
MIP-1 alpha (CCL3)	**2.23**	1.34	1.08	1.37	**2.84**	**2.28**	**2.82**
G-CSF	2.22	1.20	1.19	2.18	**3.33**	**2.59**	**3.37**
IL-23	2.09	0.84	0.75	**0.37**	1.98	1.04	1.93
MIP-1 beta (CCL4)	**2.06**	1.32	1.17	1.25	**3.60**	**2.94**	**3.67**
IL-9	**2.00**	1.06	1.05	1.06	**2.98**	1.28	**1.92**
ENA-78 (CXCL5)	1.84	0.71	0.91	1.40	1.84	1.26	1.80
TNF alpha	1.77	1.33	0.93	1.06	**2.95**	1.83	**2.64**
M-CSF	1.74	0.76	0.69	0.68	1.57	0.90	1.11
IL-1 alpha	1.67	0.77	0.57	0.55	1.90	0.99	1.61
IL-27	1.66	1.09	0.95	1.04	**3.32**	1.73	**2.28**
GRO-alpha (CXCL1)	1.52	1.39	0.54	0.90	**4.04**	**6.49**	**5.30**
MCP-3 (CCL7)	1.47	0.83	1.02	0.87	**3.34**	**5.99**	**5.42**
MCP-1 (CCL2)	1.33	0.88	1.13	0.83	**3.18**	**6.58**	**5.32**
IL-1 beta	1.28	1.05	1.03	1.26	**2.19**	1.63	**2.22**
Eotaxin	1.12	1.00	0.99	0.95	1.11	1.08	1.12
RANTES (CCL5)	1.01	1.03	0.93	0.94	1.21	1.32	1.21
IL-31	0.75	1.04	1.04	1.00	0.80	1.09	0.79
IL-28	0.69	1.05	1.01	0.93	0.74	1.15	0.87
C							
F1							
Cytokine^a^	F1V + Alu	F1:NLP	F1:NLP + Alu	V:NLP	V:NLP + Alu	F1:V:NLP	F1:V:NLP + Alu
IL-5	**123.78**	2.73	**186.62**	1.96	**11.51**	**5.96**	**319.20**
IL-13	**42.87**	2.51	**50.84**	1.31	**8.85**	**6.75**	**102.40**
IL-3	**26.14**	**5.36**	**25.97**	1.90	**11.02**	**11.45**	**51.14**
IL-17A (CTLA-8)	**23.72**	3.88	**4.74**	1.96	**10.74**	**29.02**	**42.42**
IL-4	**15.46**	3.03	**12.66**	1.55	**8.22**	**6.97**	**20.57**
IL-2	**14.83**	**3.44**	**13.71**	1.02	**3.95**	**4.48**	**13.78**
IL-22	**11.62**	**5.62**	**7.85**	2.20	**25.45**	**26.36**	**44.14**
IFN gamma	6.71	4.06	**9.06**	1.90	**19.89**	**17.28**	**39.26**
IL-6	**6.67**	1.81	**4.61**	1.12	**6.16**	**9.98**	**20.83**
GM-CSF	**6.07**	1.92	**5.59**	1.17	**5.18**	**4.51**	**13.25**
IL-18	**5.07**	3.09	**5.44**	1.56	**9.67**	**9.20**	**14.80**
IL-15	**3.32**	2.51	**2.93**	1.42	**4.39**	**4.48**	**3.26**
IP-10 (CXCL10)	2.87	1.50	1.88	1.01	**7.23**	**9.32**	**11.48**
LIF	2.65	1.23	1.59	1.15	2.78	**3.97**	**6.03**
MCP-3 (CCL7)	2.11	1.09	1.79	0.97	**4.37**	**7.46**	**7.72**
IL-12p70	2.10	1.37	2.35	1.28	**3.91**	**3.68**	**5.61**
MCP-1 (CCL2)	1.80	1.07	1.76	0.88	**4.32**	**7.81**	**7.27**
MIP-1 alpha (CCL3)	1.74	1.28	1.49	1.43	**2.41**	**2.60**	**3.25**
GRO-alpha (CXCL1)	1.64	1.59	0.72	0.93	**4.80**	**7.20**	**7.72**
ENA-78 (CXCL5)	1.62	1.52	1.86	2.41	1.47	2.21	2.51
MIP-1 beta (CCL4)	1.54	1.28	1.48	1.31	**2.73**	**3.18**	**3.82**
IL-10	1.43	1.06	1.26	0.70	2.12	2.04	**2.61**
IL-23	1.33	0.75	0.80	**0.36**	1.01	0.90	1.40
IL-27	1.32	1.08	1.33	1.05	**2.41**	2.03	**2.44**
G-CSF	1.31	1.52	1.56	2.33	2.22	**2.70**	**2.97**
M-CSF	1.30	0.60	0.70	**0.42**	0.72	1.01	0.92
TNF alpha	1.24	1.26	1.29	1.17	**2.12**	**2.12**	**2.72**
IL-9	1.15	1.02	1.20	0.98	1.16	1.10	1.19
IL-31	1.14	1.02	1.11	0.97	1.13	1.07	1.20
IL-1 alpha	1.11	0.50	0.67	**0.42**	1.16	0.88	1.38
Eotaxin	1.06	0.98	1.04	0.94	1.10	0.89	1.15
RANTES (CCL5)	1.03	1.01	0.97	1.05	1.45	**1.48**	**1.47**
IL-1 beta	0.96	0.97	1.20	1.54	**2.13**	1.83	**2.48**
IL-28	0.87	1.03	0.89	0.89	1.18	1.13	0.97

a The cytokine results are shown as the ratio to PBS (Sham), and are based on the Geometric Mean (pg/ml). Bolded (p < 0.05).

Vaccines were given 21 days apart. (data from experiment B, *n* = 6). 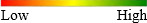

It was notable and encouraging that both the benchmark F1V vaccine and F1:V:NLP, when formulated with Alhydrogel and CpG, produced highly similar recall responses in splenocytes. Any notable differences between the two vaccines were only seen in Experiment A or B but not both, with the exception that F1:V:NLP + Alu and V:NLP + Alu splenocytes produced at least twice as much IL-10 than F1V splenocytes in both experiments. IL-10 is an anti-inflammatory cytokine that plays a role in maintaining tissue homeostasis and limiting tissue damage. Otherwise, splenocytes from both groups produced especially high levels of Th2 cytokines (IL-5, IL-13, IL-4), and elevated levels of several others including the Th1 cytokine IFN-γ; the T cell stimulant IL-2; and proinflammatory cytokines like GM-CSF and IL-6 that are mostly produced by myeloid cells.

### 3.3 The antibody response generated with the NLP platform is comparable to that of the traditional subunit F1V vaccine when Alhydrogel is included

#### 3.3.1 Antibody titers

**Figure 3:**
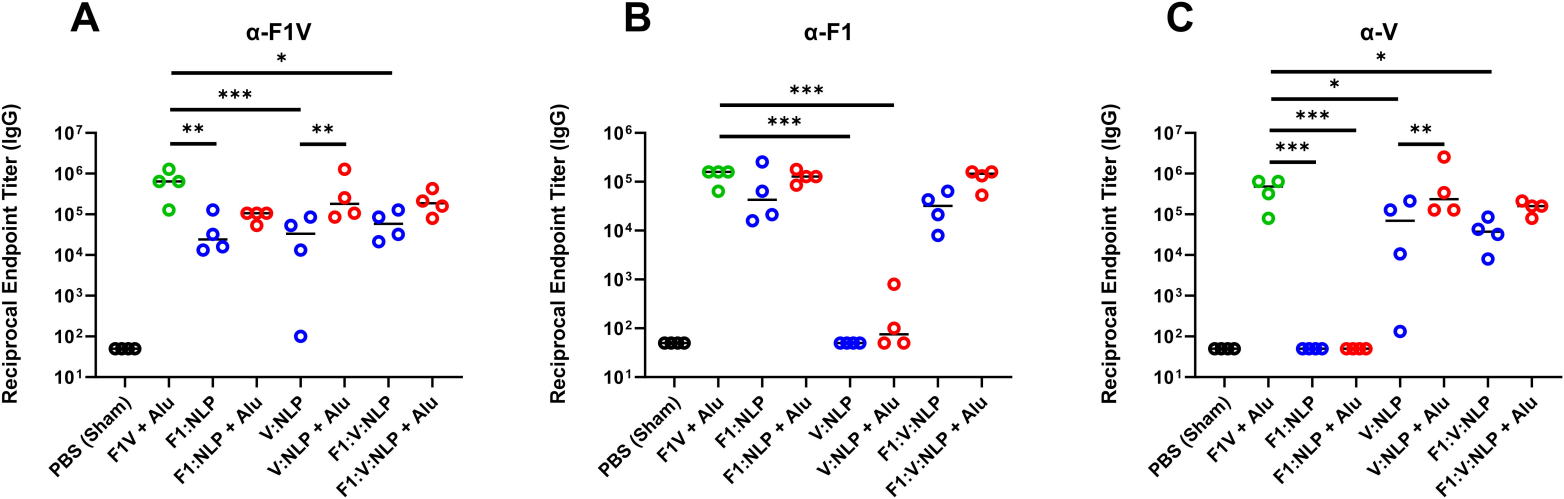
Total IgG response against **(A)** F1V, **(B)** F1, and **(C)** V, antigens at 27 days post-vaccination (pre-challenge). The F1V fusion benchmark vaccine is green. The experimental vaccine formulations without Alu are blue and those that include Alu are red. *n* = 4 animal sera per group. For ELISA, pairwise treatment groups were compared by negative binomial generalized linear mixed model. Complete results from statisitical analyses are described in **Supplementary Table 3**. * < 0.05, ** < 0.01, and *** < 0.001.

It has been previously shown that strong antibody titers, following vaccination with F1V fusion protein or F1 and/or V, are indicative of protection against *Y. pestis* aerosol challenge (35, 38, 55, 56, 71–75). Mice were vaccinated with two doses of the vaccines four weeks apart, and serum antibody titers were evaluated four weeks following the second dose. As expected, the anti-F1V total IgG titers were highest after F1V + Alu vaccination, and the NLP vaccines produced strong responses when formulated with Alu. In particular, V:NLP + Alu mice had significantly higher (*p* ≤ 0.0005) anti-F1V and anti-V titers than mice given V:NLP alone (**Figure 3**, **Supplementary Table 3**). The total anti-F1V IgG response with all NLP formulations was diminished relative to F1V vaccine, although this impairment in IgG response compared to F1V was only significant (*p* ≤ 0.005) in the groups without Alu (**Figure 3A**). The anti-F1 IgG response was similar between the F1V + Alu and F1-containing NLP groups (F1: NLP + Alu and F1:V:NLP + Alu) (**Figure 3B**). The anti-V IgG response was similar between F1V + Alu and V:NLP + Alu groups, and slightly but not significantly diminished in the F1:V:NLP + Alu group (**Figure 3C**). A similar antibody response enhanced by the presence of Alu was also observed in mice that received a boost 21 days after the prime rather than 28 (**Supplementary Table 4**).

**Figure 4:**
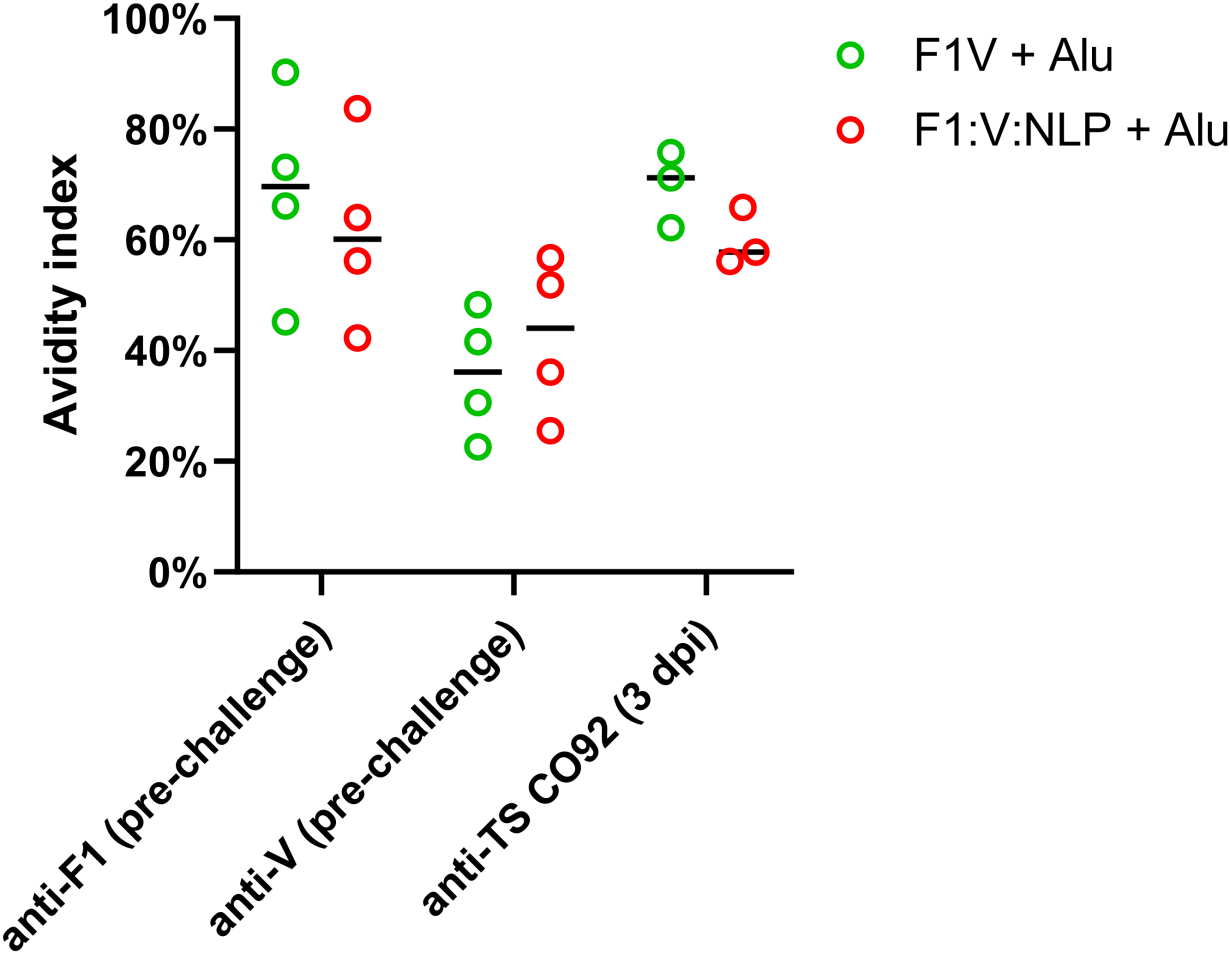
Serum samples from 28 days post-vaccination (pre-challenge) or 3 days post-infection (dpi) were measured for total serum IgG against F1, V, or TS CO92 antigens using an endpoint ELISA assay on a series of 2-fold dilutions of serum, performed with an additional incubation step with either wash buffer, or 6 M urea to disrupt weak antibody-antigen binding. The avidity index was analyzed by comparing (OD_450_ – OD_570_) values at the lowest dilution that had an (OD_450_ – OD_570_) below 1.5 in the no urea condition. Avidity index was calculated as (OD with urea/OD with wash buffer) x 100%.

To assess whether sera with similar antibody titers might differ in the strength of their antigen binding (avidity), we used urea to create chaotropic conditions and disrupt weak binding on the ELISA plates, following a method used to compare serum avidity induced by different vaccine platforms and adjuvants (76, 77). We compared the F1V + Alu and F1:V:NLP + Alu groups, as both had high total IgG titers spanning both F1 and V antigens, and found similar avidity indexes (**Figure 4**).

**Figure 5:**
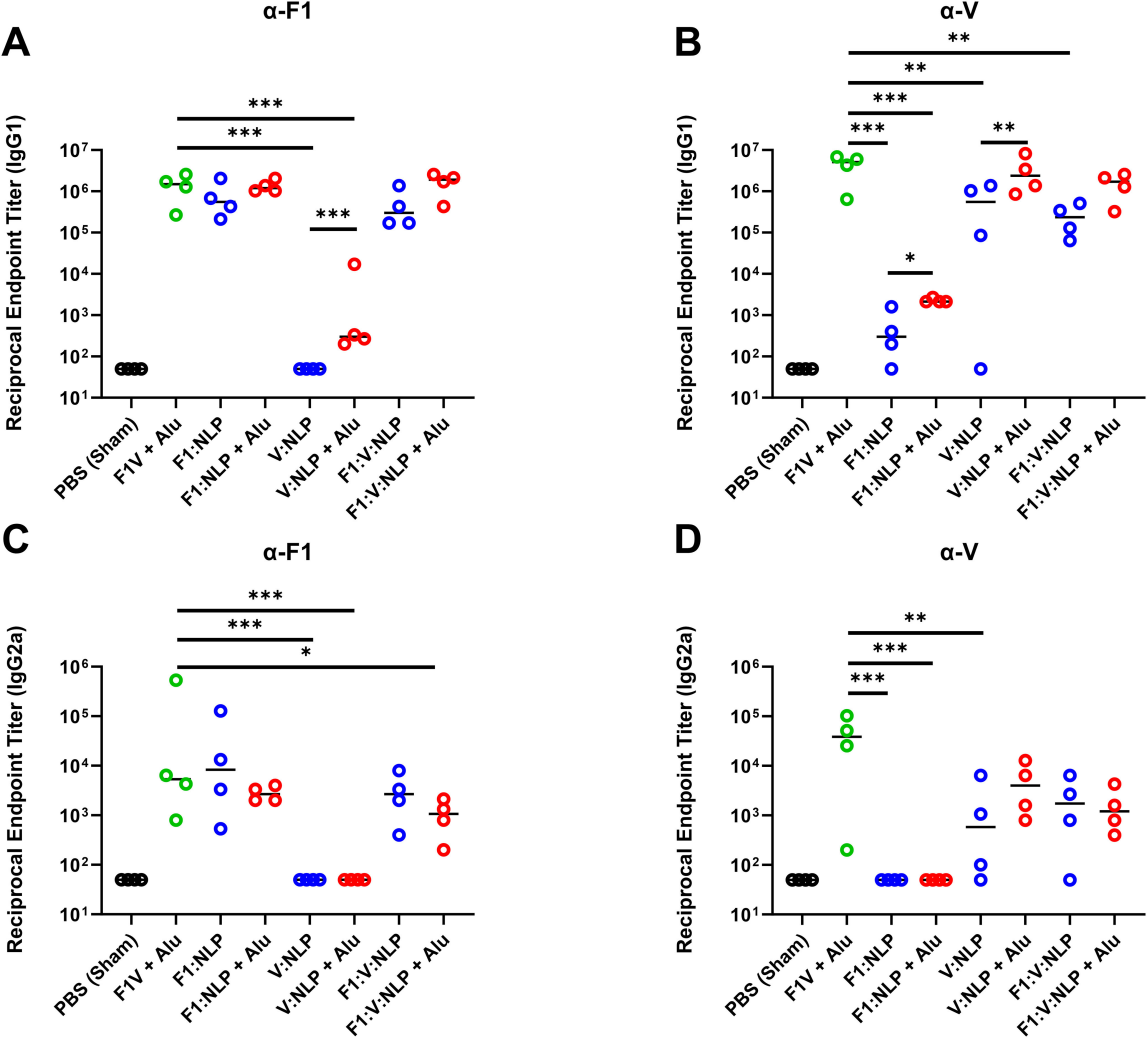
Antibody IgG1 and IgG2a subclass response against F1 **(A, C)** and V **(B, D)**, antigens at 27 days post-vaccination (pre-challenge). The F1V fusion benchmark vaccine is green. The experimental vaccine formulations without Alu are blue and those that include Alu are red. *n* = 4 animal sera per group. For ELISA, pairwise treatment groups were compared by negative binomial generalized linear mixed model. Complete results from statisitical analyses are described in **Supplementary Table 5**. * < 0.05, ** < 0.01, and *** < 0.001.

Regarding IgG1 titers against F1, only NLP formulations with Alu induced IgG1 titers at a comparably high level as F1V + Alu, although the differences between NLP formulations with and without Alu were not statistically significant (**Figure 5A**, **Supplementary Table 5**). However, for IgG1 titers against V, as with total IgG titers, V:NLP + Alu mice had significantly higher IgG1 titers (*p* < 0.01) than mice given V:NLP alone, exhibiting a 4-fold increase based on median titers or 50-fold increase based on geometric means (**Figure 5B**, **Supplementary Table 5**). Increased IgG1 with Alu was also seen when comparing F1:V:NLP and F1:V:NLP + Alu, an approximate 7-fold increase based on medians though not significant. Meanwhile for IgG2a, addition of Alu to the NLP formulations had less effect on titers, and appeared to reduce IgG2a titers against F1, leading to F1:V:NLP + Alu having significantly less anti-F1 IgG2a than the F1V + Alu vaccine (**Figures 5C, D**). This was the only significant difference in anti-F1 IgG1 or IgG2a between F1V + Alu and any F1:NLP formulations, whereas for anti-V, there were three comparisons where V:NLP formulations had significantly lower titers than F1V + Alu (**Supplementary Table 5**).

#### 3.3.2 B-cell ELISpot assays

**Figure 6:**
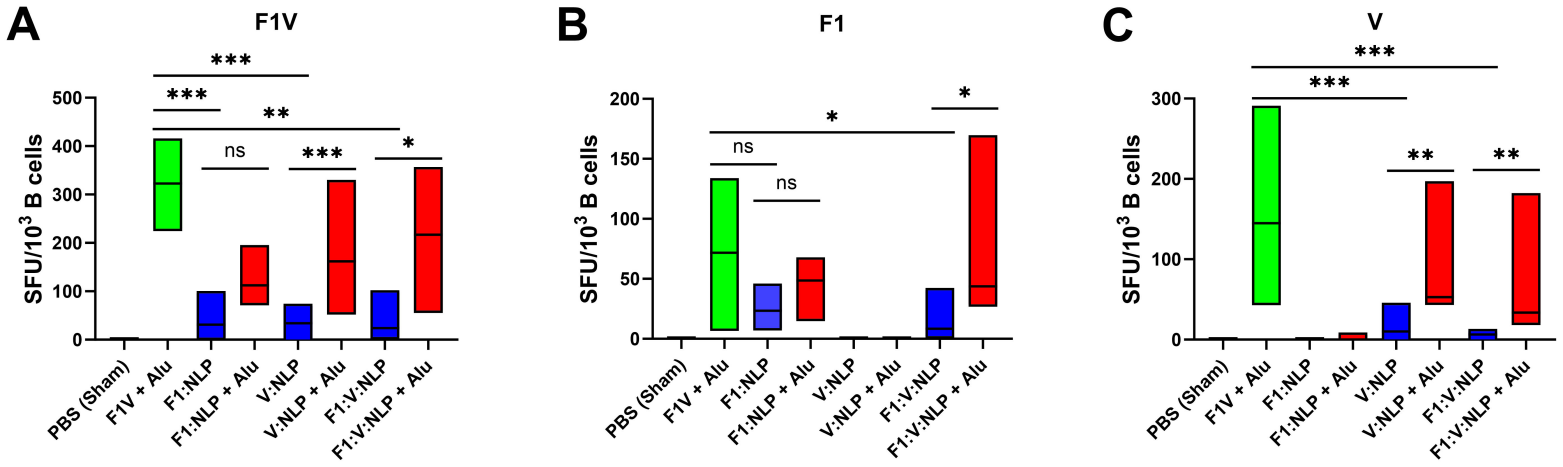
IgG1-secreting B cells detected 27 days post-vaccination after stimulations with **(A)** F1V, **(B)** F1 and **(C)** V antigens. The F1V fusion benchmark vaccine is green. The experimental vaccine formulations without Alu are blue and those that include Alu are red. For ELISpot results, data were log10 transformed prior to analysis and pairwise treatment groups were compared by linear mixed effects model. *n* = 4 mice/group. * < 0.05, ** < 0.01, and *** < 0.001.

To further assess the humoral immune response, in addition to antibody titers, we enumerated the number of antigen specific B cells in purified splenocytes approximately four weeks following the last dose of the vaccine. Similar to the cytokine response, inclusion of Alu was necessary for the induction of antigen specific B cells. The number of F1V-specific splenic B cells was highest in the F1V + Alu vaccinated mice, followed by NLP + Alu formulations (**Figure 6A**). All three NLP formulations without Alu had significantly fewer IgG1 F1V-specific B cells than the F1V + Alu vaccinated mice (*p* < 0.001), but the NLP formulations with Alu were statistically similar. The number of F1-specific B cells was also similar between F1V + Alu, F1:NLP + Alu and F1:V:NLP + Alu. The samples collected from mice vaccinated with F1:V:NLP without Alu had significantly fewer F1-specific cells than the corresponding Alu group (*p* < 0.05), while F1:NLP also had fewer cells relative to F1:NLP + Alu but this did not reach significance (**Figure 6B**). Mice vaccinated with V:NLP or F1:V:NLP without Alu had significantly fewer V-specific B cells than the corresponding groups with Alu (*p* < 0.01), which were comparable with the F1V + Alu group (**Figure 6C**), reflecting the major antibody titer differences seen in **Figure 3**.

### 3.4 The NLP vaccine platform results in a different population of splenocytes compared to the traditional protein subunit vaccine formulation

**Figure 7:**
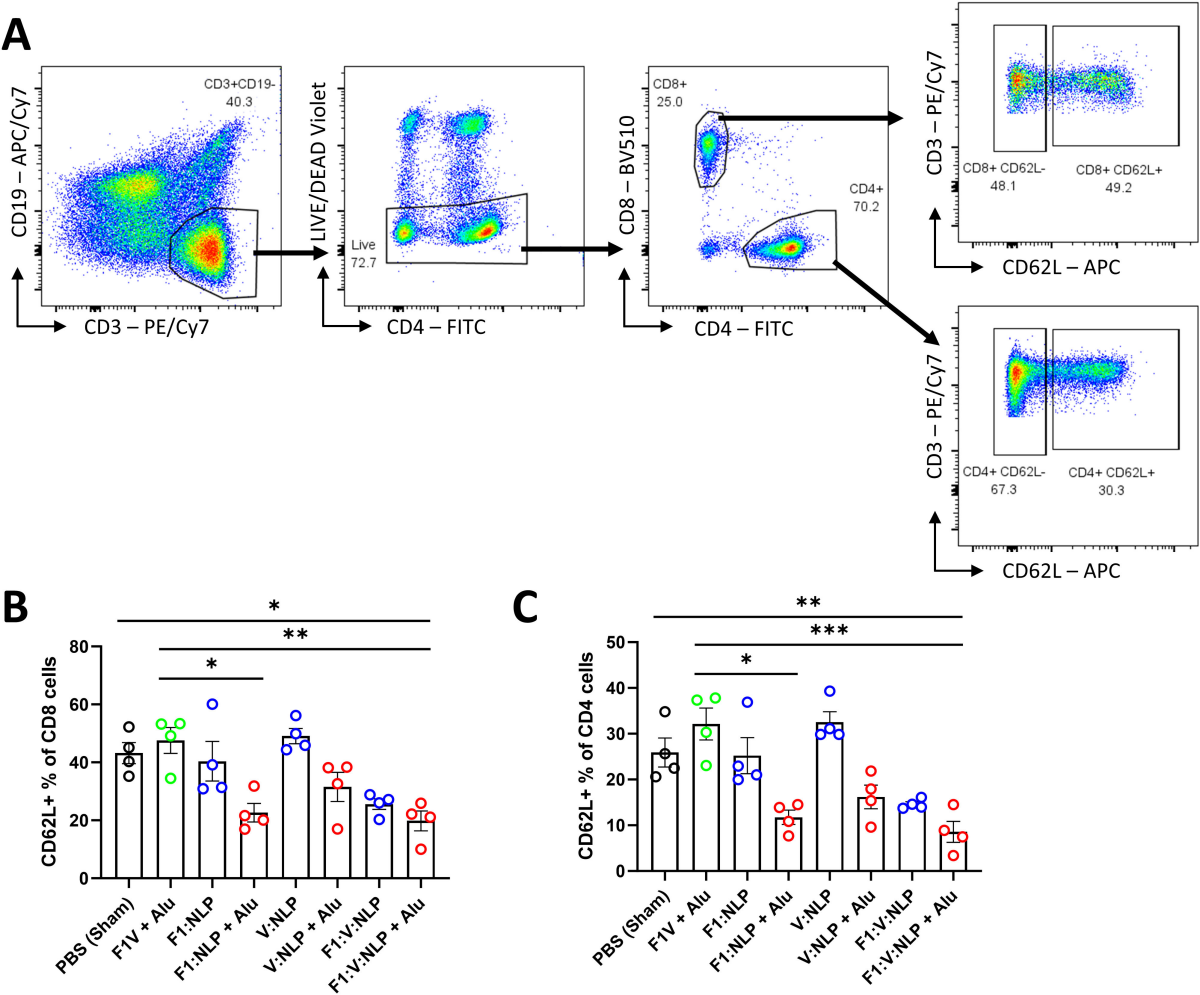
T cell populations in spleens of mice 27 days after the second vaccine dose. Splenocytes were stained for flow cytometry, fixed in 2% formaldehyde, and run on a FACSCanto II. **(A)** T cells were defined as viable, CD19-, CD3+ lymphocytes, then gated into CD8+ or CD4+ T cell populations, and defined as either positive or negative for CD62L. **(B, C)** The CD62L+ percentage of CD8 **(B)** and CD4 **(C)** T cells were compared across vaccine groups. The F1V fusion benchmark vaccine is green. The experimental vaccine formulations without Alu are blue and those that include Alu are red. Individual points represent one animal (*n* = 4 mice/group), and the columns and error bars represent mean ± SEM. Negative binomial regression was used for the flow cytometry data analysis. * < 0.05, ** < 0.01, and *** < 0.001.

**Figure 8:**
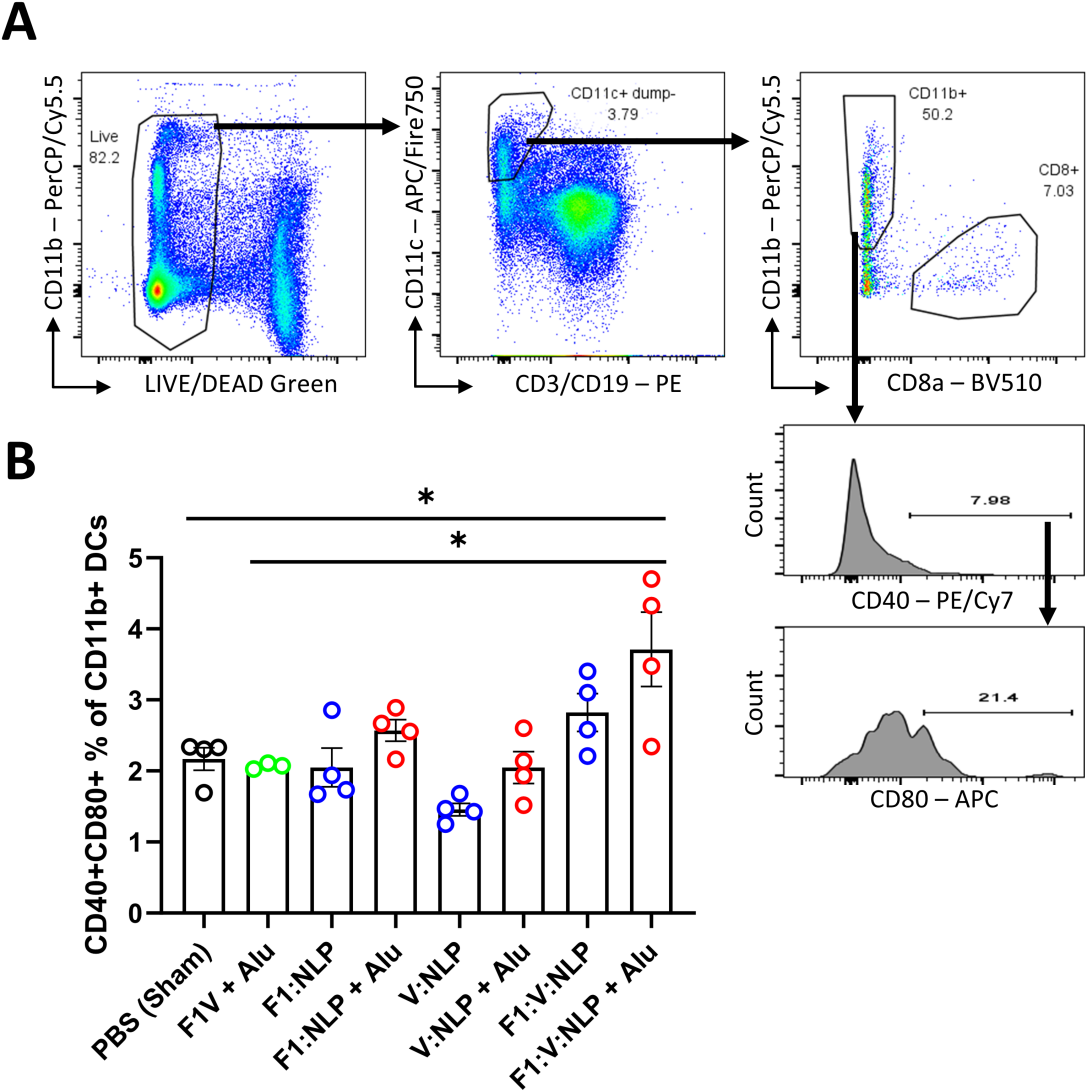
Dendritic cell populations in spleens of mice 27 days after the second vaccine dose. Splenocytes were stained for flow cytometry, fixed in 2% formaldehyde, and run on a FACSCanto II. **(A)** Dendritic cells were defined as viable, CD19-, CD3-, CD11c+ cells, and then defined as CD11b+ (direct presenting) or CD8+ (cross-presenting). CD11b+ DCs were analyzed to measure the number that upregulated costimulatory molecules CD40 and CD80. **(B)** The CD40/CD80+ double-positive percentage of CD11b+ DCs was compared across vaccine groups. The F1V fusion benchmark vaccine is green. The experimental vaccine formulations without Alu are blue and those that include Alu are red. Individual points represent one animal (*n* = 4 mice/group), and the columns and error bars represent mean and SEM. Negative binomial regression was used for the flow cytometry data analysis. * < 0.05.

To assess innate and cell-mediated immune responses induced by vaccination, we used flow cytometry to phenotype the purified splenocytes approximately four weeks after the second vaccine dose. CD62L (L-Selectin) was used to distinguish naïve and activated T cell phenotypes. On T lymphocytes, CD62L is needed for naïve cells to home to lymphoid organs, and its loss is indicative of activation, cell division and acquiring a memory or effector phenotype (78–80) (**Figure 7A**). Upregulation of costimulatory molecules CD80 and CD40 was used to distinguish activated CD11b^+^ dendritic cells (DCs) associated with antigen presentation to CD4^+^ T cells (81, 82) (**Figure 8A**). Mice vaccinated with F1V + Alu showed no reduction in CD62L on T cells relative to the PBS group, while F1:V:NLP + Alu vaccinated mice had a significant decrease in CD62L on both CD8^+^ (**Figure 7B**) and CD4+ (**Figure 7C**) T cells. In addition, among the NLP vaccine groups, F1:NLP + Alu and V:NLP + Alu showed a loss of CD62L on T cells compared to the corresponding groups without Alu, as well as the loss of CD62L compared to F1V + Alu, indicating that the combination of NLP and Alu was highly immunogenic, although the loss of CD62L was only significant in one comparison (**Figures 7B, C**). Likewise, mice vaccinated with F1V + Alu showed no upregulation of CD80 and CD40 on DCs, while F1:V:NLP + Alu vaccinated mice had a significant increase compared to PBS and F1V + Alu (**Figure 8B**). In this analysis of bulk splenocytes it appeared that more CD4 T cells, CD8 T cells, and dendritic cells had an activated phenotype following F1:V:NLP + Alu vaccination; whereas in mice vaccinated with F1V + Alu, the relative proportions of naïve and activated T cells and DCs was similar to the PBS group.

### 3.5 When formulated with Alhydrogel the NLP platform containing the F1 and V antigens offers significant protection against pneumonic plague in a mouse model of disease

**Figure 9:**
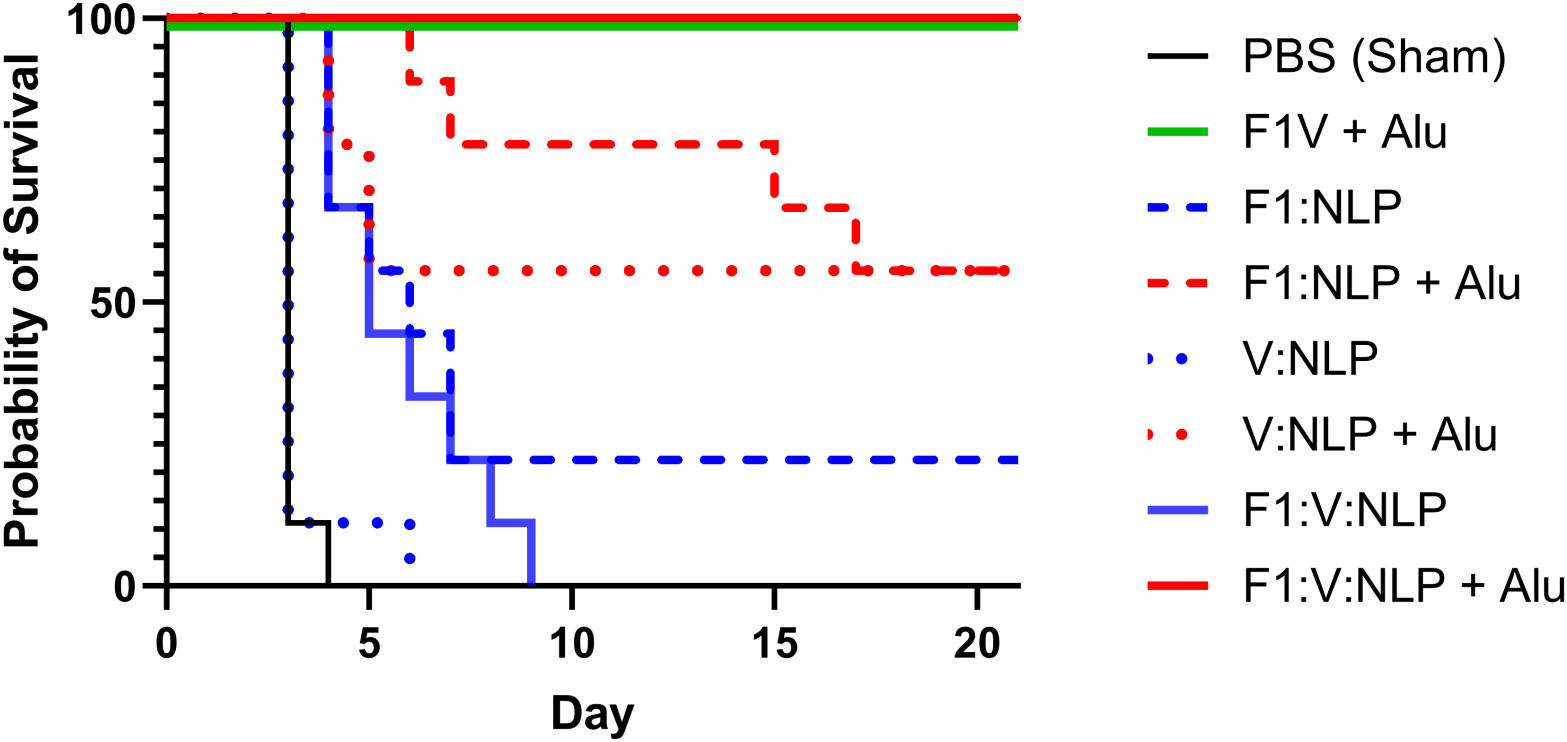
Survival curves of vaccinated and control BALB/c mice challenged with *Y. pestis* CO92. Mice (*n* = 9 mice/group) were exposed to 5.25 x 10^5^ CFU (~8 LD_50_) of *Y. pestis* CO92 via aerosol exposures. The F1V fusion benchmark vaccine is green. The experimental vaccine formulations without Alu are blue and those that include Alu are red. The mouse survival rates at selected time points were compared by Fisher exact test and the time to death or euthanasia (TTD) were analyzed by Log-rank test for the comparison against PBS group.

The F1V vaccine is highly efficacious at protecting against encapsulated *Y. pestis* and is thus an appropriate comparator to the NLP formulations. Four weeks following the last dose of the vaccine, mice were exposed to aerosolized *Y. pestis* CO92 (approximately 8 LD_50_) and monitored for 21 days. All sham (PBS) vaccinated mice succumbed to infection or were euthanized in accordance with early endpoint euthanasia criteria 4 days following challenge, and the V:NLP vaccine conferred no significant protection relative to the sham group (**Figure 9**). The F1:NLP vaccine was able to confer 22% protection while all F1:V:NLP vaccinated mice succumbed to infection within nine days following challenge. While none of the vaccines without Alu resulted in significant protection, addition of Alu to the vaccine formulations drastically increased vaccine efficacy conferring 56% protection to both F1:NLP + Alu and V:NLP + Alu vaccinated mice (*p* = 0.029 compared to negative control mice). Mice vaccinated with either the F1V + Alu or the F1:V:NLP + Alu were fully protected for the duration of the study (*p* < 0.0001 compared to negative control mice) (**Figure 9**).

### 3.6 Bacterial burden at day 3 post-infection is associated with protection from disease independent of the vaccine platform employed

**Figure 10:**
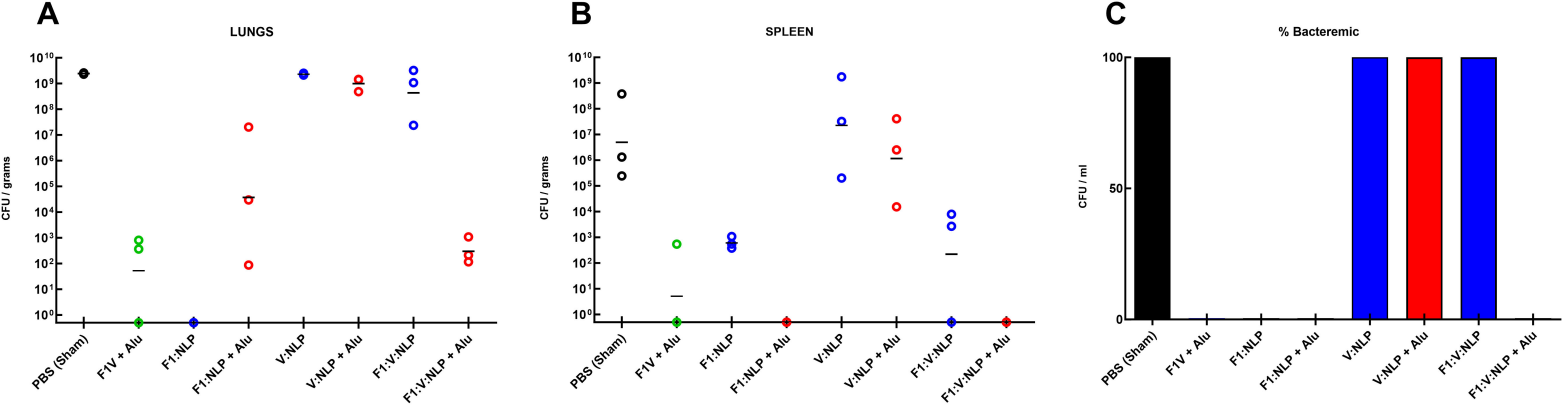
The recovery of bacteria as determined by CFU counts from **(A)** lung, **(B)** spleen and **(C)** blood of BALB/c mice three days post-challenge with *Y. pestis* CO92. The left axis represents CFU/g (Lung and Spleen) or CFU/mL (Blood). The F1V fusion benchmark vaccine is green. The experimental vaccine formulations without Alu are blue and those that include Alu are red. The individual points represent one animal (*n* = 3 mice/group), and the baseline points indicate the remaining survivors with no detectable CFU. The horizontal lines are the geometric mean. The limits of detection were approximately 100 CFU/mL (blood) and 5 CFU/g (organs).

Bacterial burden was evaluated in vaccinated mice, three days post challenge, to assess the impact of bacterial growth in the lungs and dissemination to the blood and spleen (**Figure 10**, **Supplementary Table 6**). Low bacterial burden was observed in mice vaccinated with F1V + Alu and F1:V:NLP + Alu. The bacterial burdens observed in the cohorts receiving these two most-protective vaccines were statistically indistinguishable from each other when comparing bacterial burden in lungs (**Figure 10A**) and spleens (**Figure 10B**) (*p* = 0.94 and *p* = 0.19, respectively for lungs or spleens), and there was no detectable bacteria in blood in any mouse in either cohort (**Figure 10C**). Surprisingly, the F1:NLP vaccinated mice had no detectable CFUs in the lungs; however, those same mice had a detectable bacterial burden in the spleen, indicative of bacterial dissemination (**Figure 10**). The F1:NLP + Alu vaccinated mice had a moderate to high bacterial burden in the lungs, but no detectable CFUs in the spleen. This is in contrast with the two most protective vaccines, F1V + Alu and F1:V:NLP + Alu, which had low to no detectable CFUs in the lungs and spleen, respectively. The addition of Alu had varying impacts on the lung bacterial burden depending upon the vaccine antigens being evaluated. In the case of the F1:V:NLP, the addition of the Alu resulted in at least a 6-log reduction of bacteria in the lungs (*p* < 0.0001). As depicted in **Figure 10B**, the addition of Alu to the vaccine formulations decreased bacterial dissemination to the spleens in two experimental vaccines F1:NLP (*p* < 0.0001) and F1:V:NLP (*p* = 0.029). The addition of Alu did enhance the bacterial clearance associated with the V:NLP vaccine in the lungs (*p* = 0.017) but did not decrease dissemination to or clearance from the spleen in a statistically significant manner (*p* = 0.076). Mice vaccinated with V:NLP, F1:V:NLP, and V:NLP + Alu also had recoverable bacteria in the blood (**Figure 10C**), even though V:NLP + Alu conferred 56% protection (**Figure 9**).

### 3.7 Suppression of the “cytokine storm” at day 3 post-infection is associated with protection from disease independent of the vaccine platform employed

The ability of vaccines to mitigate cytokine storm is indicative of protective efficacy following infection. Accordingly, we analyzed the cytokine profile in the lungs and spleens of mice three days following challenge. Results are expressed as fold changes (**Table 4**) and concentrations (pg/mL; **Supplementary Table 7**). Lower fold changes representing a more controlled cytokine response following challenge were consistent with reduced bacterial burden in those organs (**Figure 10**). Specifically, vaccine formulations that resulted in lower levels of IL-6, IL-22, IFN-γ, IL-17A G-CSF, GM-CSF, TNF-α, CCL3, IL-1β, CXCL1, IL-1α, CCL4, IL-12p70, and IL-27 in the lung afforded protection from challenge. A high bacterial burden in the lungs of mice vaccinated with V:NLP, V:NLP + Alu and F1:V:NLP was associated with a similar uncontrolled cytokine response in the lungs, with similar cytokine levels compared to the sham vaccinated mice. The F1:NLP vaccinated mice which had no detectable CFUs in the lungs also had cytokine levels that were drastically lower relative to the control group, while the F1:NLP + Alu vaccinated mice which had a moderate amount of CFUs in the lungs also had a less pronounced reduction in cytokine levels. Similar to the cytokine response in the lungs, lower bacterial burden in the spleens of mice vaccinated with the F1 inclusive vaccines was associated with a decreased cytokine response relative to sham vaccinated mice (**Table 4**, **Supplementary Table 7**). The two most protective vaccines, F1V + Alu and F1:V:NLP + Alu, had the lowest induction of cytokines in both lungs and spleens, with F1:V:NLP + Alu vaccinated mice showing the lowest levels of cytokine induction.

**Table 4:** Fold change relative to PBS in cytokine responses in lung and spleen homogenates from mice three days post-challenge with *Y. pestis* CO92.

Lung							
Cytokine^a^	F1V + Alu	F1:NLP	F1:NLP + Alu	V:NLP	V:NLP + Alu	F1:V:NLP	F1:V:NLP + Alu
IL-5	**8.928**	1.018	**16.126**	2.434	**26.004**	1.064	3.116
Eotaxin	1.740	1.191	1.215	1.274	1.014	0.634	0.930
IL-28	1.484	1.595	1.533	0.663	1.322	0.872	1.563
RANTES (CCL5)	1.049	0.954	1.130	1.115	1.172	0.893	0.967
IFN alpha	1.011	1.000	1.195	1.197	**1.908**	1.187	1.000
IL-31	1.000	1.000	0.685	1.000	0.458	0.434	1.000
IL-3	1.000	1.000	0.826	1.000	**0.476**	0.734	1.000
IL-2	0.893	0.722	0.769	1.032	0.880	0.698	0.575
IL-23	0.784	0.930	1.083	0.989	1.562	1.173	0.743
IL-4	0.678	0.442	3.848	1.548	1.517	0.951	0.854
IL-15	0.675	0.571	0.805	1.300	1.620	0.799	**0.297**
ENA-78 (CXCL5)	0.587	0.605	0.281	1.183	1.512	0.569	0.589
IL-9	0.494	**0.353**	0.719	1.426	1.157	0.844	**0.310**
IL-10	0.477	0.507	0.641	1.301	0.869	0.678	0.465
M-CSF	0.454	0.405	0.775	1.595	2.383	1.142	**0.199**
IP-10 (CXCL10)	0.348	0.323	0.718	1.320	1.345	0.896	**0.237**
IL-13	0.317	0.491	1.230	1.497	1.468	0.883	1.134
IL-27	**0.224**	**0.220**	0.337	1.556	0.802	0.591	**0.163**
MCP-3 (CCL7)	0.210	0.121	0.503	1.032	1.182	0.763	**0.068**
IL-12p70	**0.170**	**0.203**	0.694	1.050	1.431	0.829	**0.170**
MIP-1 beta (CCL4)	**0.108**	**0.091**	0.351	1.587	1.412	0.988	**0.078**
IL-1 alpha	**0.085**	**0.066**	0.257	1.048	1.137	0.800	**0.055**
GRO-alpha (CXCL1)	**0.080**	**0.046**	0.294	2.275	2.537	1.418	**0.037**
IL-1 beta	**0.049**	**0.041**	**0.187**	1.337	1.550	0.947	**0.030**
MIP-1 alpha (CCL3)	**0.048**	**0.040**	0.216	1.195	1.682	0.927	**0.031**
MCP-1 (CCL2)	0.039	**0.023**	0.122	1.836	1.224	0.450	**0.004**
TNF alpha	**0.021**	**0.015**	**0.113**	1.011	0.749	0.576	**0.013**
GM-CSF	**0.021**	**0.022**	**0.100**	0.904	0.999	0.548	**0.013**
G-CSF	**0.019**	**0.014**	0.090	2.162	1.569	1.143	**0.005**
IL-17A (CTLA-8)	**0.017**	**0.004**	0.209	1.552	1.215	1.160	**0.003**
IFN gamma	**0.013**	**0.015**	0.133	1.289	1.105	0.242	**0.006**
IL-22	**0.007**	**0.013**	0.239	1.136	1.384	0.586	**0.005**
IL-6	**0.006**	**0.004**	**0.021**	1.943	1.414	0.519	**0.002**
Spleen							
Cytokine^a^	F1V + Alu	F1:NLP	F1:NLP + Alu	V:NLP	V:NLP + Alu	F1:V:NLP	F1:V:NLP + Alu
RANTES (CCL5)	2.716	2.745	1.063	1.103	1.135	1.131	1.633
IL-4	1.772	1.288	0.870	2.104	4.657	0.365	1.155
IL-5	1.318	1.000	1.000	1.083	**9.810**	1.000	1.000
IL-9	1.216	1.000	1.000	1.366	1.236	1.000	1.000
IL-15	1.025	0.665	0.664	1.723	2.256	0.664	0.664
IFN alpha	1.000	1.000	1.000	1.085	1.000	1.000	1.000
IL-31	1.000	1.000	1.000	1.000	1.000	1.000	1.000
IL-3	1.000	1.000	1.000	1.000	1.000	1.000	1.000
IL-23	1.000	1.000	1.000	1.000	1.000	1.000	1.000
M-CSF	0.979	1.250	0.430	1.777	1.792	0.281	1.266
ENA-78 (CXCL5)	0.914	0.525	0.954	0.904	1.201	1.794	0.393
GM-CSF	0.901	0.627	0.627	2.472	2.028	0.627	0.627
IL-13	0.752	0.522	0.522	1.963	1.312	0.522	0.522
IL-10	0.748	**0.278**	0.655	1.577	2.394	**0.316**	0.542
IL-28	0.648	1.452	1.857	3.230	**7.356**	0.899	0.576
IL-2	0.624	0.621	0.621	1.975	1.882	0.621	0.621
IL-27	0.470	0.391	**0.239**	2.260	2.083	**0.202**	0.257
IL-17A (CTLA-8)	0.384	0.454	0.454	2.980	0.725	0.454	0.454
IL-12p70	0.326	**0.199**	**0.197**	1.899	2.934	**0.201**	**0.107**
MIP-1 beta (CCL4)	0.320	0.629	0.461	2.109	1.036	0.379	0.502
TNF alpha	0.259	0.191	0.191	1.520	0.936	0.191	0.191
IL-22	0.194	0.184	0.184	2.406	0.530	0.184	0.184
Eotaxin	**0.149**	**0.186**	**0.156**	1.440	0.509	0.231	**0.216**
IP-10 (CXCL10)	**0.125**	**0.144**	**0.093**	1.208	1.517	**0.099**	**0.114**
MIP-1 alpha (CCL3)	**0.122**	**0.166**	**0.102**	2.223	0.826	**0.091**	**0.137**
IL-1 beta	**0.120**	**0.135**	**0.152**	1.736	0.673	**0.188**	**0.086**
IL-1 alpha	**0.059**	**0.045**	**0.055**	0.856	0.669	**0.093**	**0.025**
MCP-1 (CCL2)	0.042	**0.024**	**0.024**	2.015	1.841	**0.024**	**0.024**
MCP-3 (CCL7)	**0.039**	**0.040**	**0.035**	3.373	0.875	**0.069**	**0.034**
IL-6	0.028	0.028	**0.012**	3.075	0.919	0.053	**0.012**
IFN gamma	**0.026**	**0.026**	**0.006**	2.821	4.438	**0.007**	**0.007**
GRO-alpha (CXCL1)	**0.015**	**0.014**	**0.030**	1.185	0.353	0.227	**0.009**
G-CSF	**0.009**	**0.009**	**0.015**	1.870	0.482	0.164	**0.009**

a The cytokine results are shown as the ratio to PBS (Sham) vaccinated mice, and are based on the Geometric Mean (pg/mL).

Bolded (*p* < 0.05).

(data from experiment A, *n* = 3). 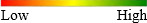

### 3.8 Antibody titers three days after exposure to aerosolized *Y. pestis* were reflective of protection and demonstrated the importance of Alhydrogel

To further correlate the protective efficacy of the vaccines to the immune response we evaluated total IgG antibody titers 3 days post challenge directed against irradiated whole cell CO92 TS. The two most protective vaccine formulations, F1V + Alu and F1:V:NLP + Alu, also had the highest total IgG antibody response (**Table 5**, **Supplementary Table 8**). These formulations both produced strong IgG1 but not IgG2a endpoint titers and were similar in terms of avidity (**Figure 4**). Surprisingly the F1:NLP + Alu formulation also produced a robust total IgG and IgG1 response yet was only partially protective. Furthermore, while the V:NLP + Alu vaccinated group achieved the same level of partial protection (56% survival) as F1:NLP + Alu, it had negligible anti-CO92 TS titers. None of the vaccine formulations induced an IgG2a response against whole cell *Y. pestis* CO92 TS (**Table 5**).

**Table 5:** Antibody titers three days post-challenge with *Y. pestis* CO92.

Vaccine Groups ^b^	Anti-CO92 TS					
	IgG Titer ^a^		IgG1 Titer ^a^		IgG2a Titer ^a^	
PBS (Sham)	50	(1.00)	58	(1.17)	50	(1.00)
F1V + Alu	17,418	(1.36)	137,159	(1.71)	63	(1.26)
F1:NLP	3,200	(1.84)	25,600	(1.31)	50	(1.00)
F1:NLP + Alu	13,788	(1.08)	(274,318)	(1.54)	50	(1.00)
V:NLP	50	(1.00)	50	(1.00)	50	(1.00)
V:NLP + Alu	50	(1.00)	159	(1.26)	50	*(1.00)
F1:V:NLP	317	*(1.26)	2,263	*(1.41)	50	*(1.00)
F1:V:NLP + Alu	20,319	(1.26)	117,579	(1.23)	50	(1.00)

a Values represent total IgG, IgG1 and IgG2a geometric mean with geometric standard error (GSE).

b *n* = 3 animal sera per group with the exception of the following groups annotated as * where *n* = 2. 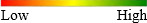

Consistent with previous data, inclusion of Alu was critical for the induction of a robust antibody response. For total IgG and IgG1, F1:NLP + Alu and F1:V:NLP + Alu had significantly higher anti-CO92 TS titers than F1:NLP and F1:V:NLP without Alu. The majority of the anti-CO92 TS titer can be attributed to antibodies against the F1 capsule; however, V:NLP + Alu did induce low but detectable anti-CO92 TS IgG1 titers (100-400), while any antibodies induced by V:NLP was undetectable (**Table 5**).

## 4 Discussion

An effective vaccine against *Y. pestis* is urgently needed, and it would be beneficial if a vaccine candidate were based on a subunit platform that could be consistently manufactured and adapted quickly for use with different antigens and adjuvants to protect against diverse pathogens. In this study, we compared the benchmark vaccine (F1V + Alu) with a combination of *Y. pestis* F1 and V antigens delivered using the NLP platform. Both vaccines were administered in two doses, 21 or 28 days apart, and demonstrated 100% protection in a mouse model of aerosol challenge with *Y. pestis* CO92 strain.

NLPs are biological nanoparticles that exhibit no acute or long-term toxicity in rodent models (57). NLPs enhance immune responses to antigens, including soluble proteins, as well as adjuvants (57, 63). Furthermore, NLPs can facilitate antigen cross-presentation, effectively promoting dendritic cells (DCs) acquisition of exogenous antigens for presentation on MHC class I molecules and activation of CD8^+^ T-cells (83). Importantly, a path to NLP scale-up for medical countermeasure applications, including vaccines, is possible using existing technologies and approaches routine for other types of nanoparticles (e.g., liposomes). Future studies will focus on NLP scale-up and manufacturability, while also leveraging additional approaches to antigen conjugation (e.g., click chemistry) (83).

Several combinations of *Y. pestis* antigens and adjuvants were assessed in combination with the NLP platform, which can be easily complexed with immunogenic proteins in their native form. Mice were completely protected against a lethal pneumonic plague challenge after NLP vaccination with just two *Y. pestis* proteins, F1 and V, in addition to adjuvants CpG and Alu (**Figure 9**). Inclusion of Alu was key to immunogenicity and protection. This could also be seen when vaccines including only one antigen, F1 or V, provided 55% protection with Alu, and 0%-22% protection without Alu. This is an interesting observation given that Alu tends to promote a Th2-type immune response, associated largely with the production of antibodies (84). While this is beneficial for many vaccines, it may be less effective for diseases where a strong Th1-type (cell-mediated) response is needed. Future studies are underway to further define the role of Alu and CpG in promoting protection against a lethal pneumonic plague challenge.

F1:V:NLP + Alu also blocked bacterial replication and/or facilitated bacterial clearance as effectively as the benchmark vaccine (**Figure 10**). F1:V:NLP alone blocked bacterial dissemination to or replication in the spleen but had almost no effect on the CFU count in the lungs at 3 days post-infection, indicating that Alu was needed for quick immune mobilization to block the pre-inflammatory phase of pneumonic plague. This result was corroborated by cytokine data: mice vaccinated with F1:V:NLP with or without Alu had similarly low levels of inflammatory cytokines in the spleen, but mice vaccinated with F1:V:NLP + Alu had far lower inflammatory cytokines in the lungs than mice vaccinated with F1:V:NLP alone. NLP vaccines containing both F1 and V, and NLP vaccines containing just F1, were similar by these metrics (bacteria and cytokines in tissue at 3 days post-infection), but those with both F1 and V were more protective against fatal disease. Importantly, when both the lung and the spleen bacterial burden are evaluated, the two most protective vaccines not only greatly suppressed lung colonization, but they also prevented systemic dissemination.

In our model, serum antibody titers against *Y. pestis* antigens correlate best with protective efficacy of a vaccine, as has been previously reported (38, 75). In the current study, when looking at serum taken approximately four weeks after the second vaccine dose, F1:V:NLP + Alu and the benchmark F1V + Alu vaccine induced similar antibody levels against F1 and V. A similar trend was observed for antibody levels against irradiated whole cells of the CO92 strain used for the challenge. Regarding antibody avidity, F1V + Alu and F1:V:NLP + Alu were not significantly different, and both had higher avidity against F1 and CO92 than against V. Not surprisingly, the NLP vaccines containing Alu also induced higher antibody titers than the corresponding vaccines without Alu. These trends were also seen when looking at the number of splenocytes from vaccinated mice that could be induced to produce IgG1 by *ex vivo* stimulation with *Y. pestis* antigens. Finally, the importance of Alu in the vaccine formulation was most notable when looking at cytokine production in *ex vivo* stimulated splenocytes, reflecting induction of T cell immunity. Splenocytes from mice vaccinated with NLPs without Alu showed minimal stimulation, with cytokine levels reaching up to ~13-fold higher than the PBS group. However, NLP vaccines containing Alu triggered a much stronger cytokine response in stimulated splenocytes (> 100- to > 1,000-fold increases), comparable to or surpassing the F1V + Alu benchmark vaccine.

The comparison between the F1V + Alu and NLP-based vaccines highlights differences in IgG subclass responses and antibody functionality. Both vaccines produce similar levels of IgG1, which is associated with Th2-type immune responses, but the NLP + Alu vaccine generates lower levels of IgG2a compared to the F1V + Alu vaccine. IgG2a is typically linked to Th1-type immunity, which is crucial for combating intracellular pathogens (25). However, antibody effectiveness is influenced not only by subclass but also by the glycosylation state of the antibody’s Fc domain, which affects immune effector functions like complement activation and Fc receptor binding (85). Vaccine composition and regimens can shape both subclass distribution and glycosylation profiles, potentially altering antibody functionality (86, 87). While the F1V + Alu vaccine appears to induce a stronger IgG2a response, the NLP-based vaccine may compensate with antibodies that have optimized glycosylation or different epitope specificities as well as altered dynamics of B cell clonal selection, potentially enhancing their ability to control early infection. This underscores the importance of considering both the quantity and quality of antibody responses when evaluating vaccine efficacy.

Interestingly, the differences in IgG subclass responses and antibody functionality did not impair the ability of splenic T cells from vaccinated mice to produce cytokines and did not influence protective efficacy since F1:V:NLP + Alu was 100% protective. Although both F1V + Alu and F1:V:NLP + Alu provided complete protection, the F1:V:NLP + Alu vaccine exhibited more effective cytokine regulation, with lower cytokine fold changes following challenge with *Y. pestis*. This suggests that at higher challenge doses, the NLP formulation could potentially surpass the F1V chimeric protein by better controlling the cytokine storm. In addition, the NLP-based vaccine strategy may offer a key advantage over F1V + Alu by enhancing overall immunogenicity, such as through a “depot effect” prolonging the presence of antigen at the injection site which can be sampled and transferred to draining lymph nodes. This is reflected in the greater reduction of CD62L expression on splenic T cells, increased upregulation of costimulatory markers on splenic dendritic cells, and higher cytokine production by splenocytes stimulated *ex vivo*. The flow cytometry phenotyping of splenic T cells and DCs is particularly relevant to the immunogenicity of the vaccine formulation, since it is an analysis of all splenocytes rather than identifying those specific for F1 or V antigens. We have demonstrated that the NLP vaccine platform can offer comparable protection against pneumonic plage as conventional vaccine formulations. Protection against pneumonic plague is challenging, and generation of a strong mucosal immune response would most likely minimize lung colonization and expedite bacterial clearance (88, 89). The NLP platform is amenable to various routes of administration, including intranasal instillation (90). Future efforts may focus on exploring a subcutaneous “prime” followed by an intranasal “pull” to the NLP vaccination regimen to amplify the immune response at the pulmonary portal of entry. Additionally, this platform is well suited for the development of multi-pathogen vaccines which are a priority to both public health and biodefense research communities to protect individuals from greater numbers of microbial pathogens with fewer vaccine administrations (91).

## Data Availability

The original contributions presented in the study are included in the article/**Supplementary Material**. Further inquiries can be directed to the corresponding author/s.

## Supplementary material

Supplementary Figure 1 SDS-PAGE analysis of antigens. Recombinant F1 and V (5μg) were analyzed by SDS-PAGE (stained with Coomassie Blue) to evaluate apparent molecular weight and purity.
