# Supplementary material for: Nanolipoprotein particle (NLP) vaccine confers protection against Yersinia pestis aerosol challenge in a BALB/c mouse model
Source: Front Immunol. 2025 Jun 26;16:1603710. doi: 10.3389/fimmu.2025.1603710 (PMC12241055; doi:10.3389/fimmu.2025.1603710)
Supplement: Supplementary Figure 1 — SDS-PAGE analysis of antigens. Recombinant F1 and V (5μg) were analyzed by SDS-PAGE (stained with Coomassie Blue) to evaluate apparent molecular weight and purity. [file Presentation1.pptx]

## Slide 1
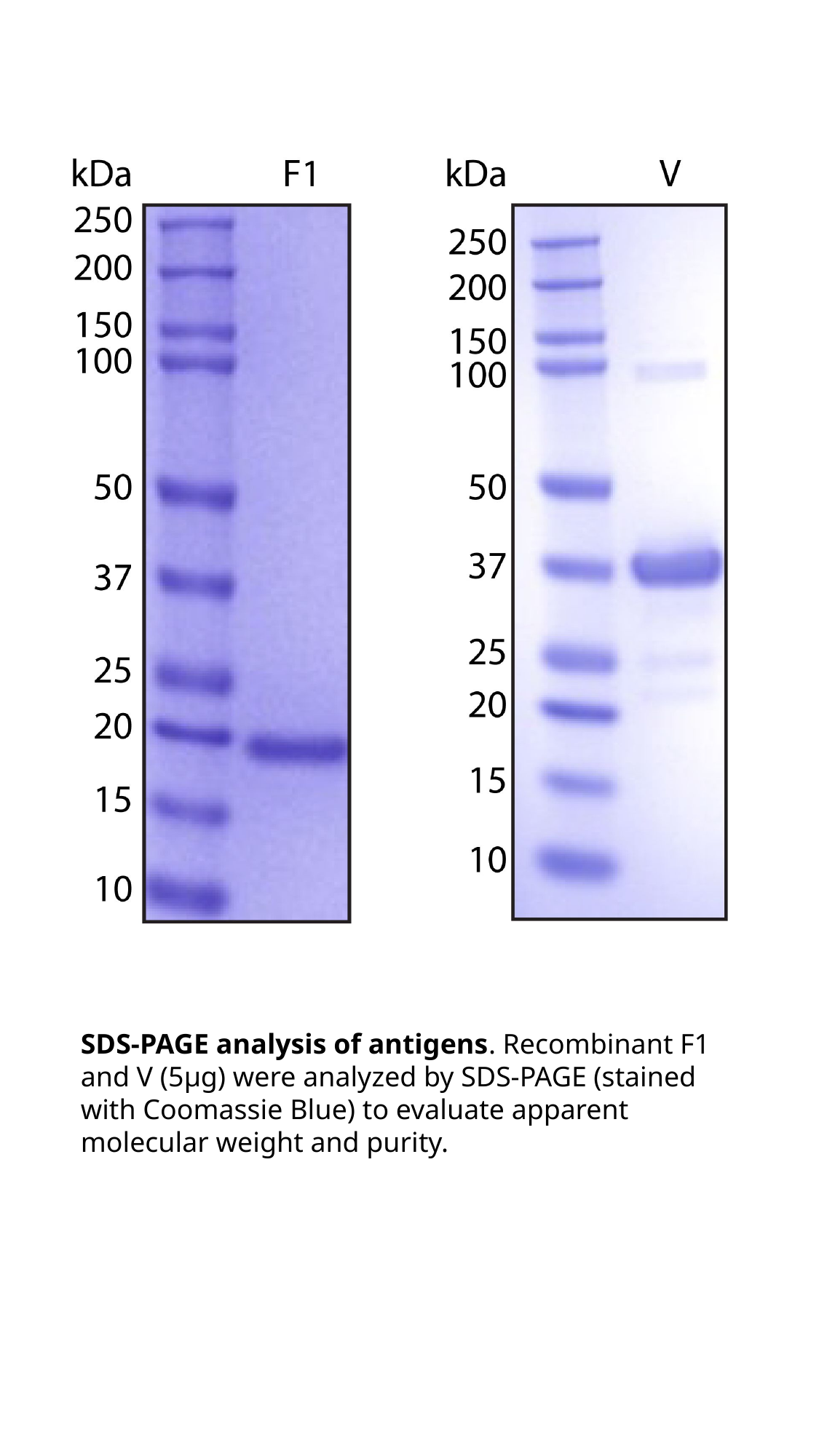

SDS-PAGE analysis of antigens. Recombinant F1 and V (5μg) were analyzed by SDS-PAGE (stained with Coomassie Blue) to evaluate apparent molecular weight and purity.
